# Cord Serum Neonicotinoid-Related Biomarkers and Neurodevelopment From Early to Middle Childhood

**DOI:** 10.1001/jamanetworkopen.2026.43481

**Published:** 2026-11-11

**Authors:** Abir Nagata, Akemi Okumura, Nagahide Takahashi, Yoko Nomura, Taeko Harada, Toshiki Iwabuchi, Tomoko Nishimura, Kenji J. Tsuchiya

**Affiliations:** 1 Research Center for Child Mental Development, Hamamatsu University School of Medicine, Hamamatsu, Japan; 2 United Graduate School of Child Development, The University of Osaka, Kanazawa University, Hamamatsu University School of Medicine, Chiba University, and University of Fukui, Suita, Japan; 3 Department of Developmental Disorders, National Center for Neurology and Psychiatry, Tokyo, Japan; 4 Queens College and Graduate Center, City University of New York, New York, New York; 5 Department of Psychiatry, Icahn School of Medicine at Mount Sinai, New York, New York; 6 Graduate School of International Cultural Studies, Tohoku University, Sendai, Japan

## Abstract

**Importance:**

Despite widespread neonicotinoid insecticide use, evidence on prenatal exposure and neurodevelopment beyond early childhood remains limited.

**Objective:**

To examine whether cord serum concentrations of the neonicotinoid-related biomarkers dinotefuran and desmethyl-acetamiprid (dm-ACE) are associated with domain-specific neurodevelopment from infancy through middle childhood.

**Design, Setting, and Participants:**

This prospective cohort study included individuals randomly selected from eligible Hamamatsu Birth Cohort for Mothers and Children Study participants born between December 2007 and March 2012 in Japan; assessments spanned ages 10 months to 9 years. Data were analyzed between October 2025 and September 2026.

**Exposures:**

Cord serum dinotefuran and dm-ACE concentrations measured at birth and categorized into quartiles.

**Main Outcomes and Measures:**

Outcomes were standardized scores for 5 Mullen Scales of Early Learning (MSEL) domains at ages 10 to 32 months and 4 Wechsler Intelligence Scale for Children, Fourth Edition (WISC-IV) indices at age 9 years. Associations were examined using linear regression and expressed as mean MSEL or WISC-IV differences in SD units at quartile 3 (Q3) and Q4 concentrations (combined Q1-Q2 was the reference).

**Results:**

Among 200 individuals, 102 (51.0%) were male and 13 (6.5%) were born preterm; 185 (92.5%) to 195 (97.5%) were included in age-specific MSEL analyses and 178 (89.0%) in WISC-IV analyses. Dinotefuran groups included 96 individuals (48.0%) in Q1 and Q2, 47 (23.5%) in Q3, and 57 (28.5%) in Q4. dm-ACE groups included 82 (41.0%) in Q1 and Q2, 63 (31.5%) in Q3, and 55 (27.5%) in Q4. Early childhood associations across neurodevelopmental scale scores varied by age and concentration group, and there were no associations after false discovery rate (FDR) correction. At age 9 years, Q3 dinotefuran concentration was associated with lower WISC-IV Processing Speed Index scores compared with Q1 to Q2 scores (mean difference, −0.50 [95% CI, −0.81 to −0.20] SDs; *q* = .01), and Q4 dm-ACE concentration was associated with lower Perceptual Reasoning Index scores (mean difference, −0.47 [95% CI, −0.81 to −0.14] SDs; *q* = .03).

**Conclusions and Relevance:**

In this cohort study of infants and children in Japan, small differences in some neurodevelopmental scale scores in early childhood were observed in association with dinotefuran and dm-ACE cord serum concentrations, but there were no associations after FDR correction. At age 9 years, dinotefuran was associated with lower processing speed and dm-ACE with lower perceptual reasoning. Overall, these findings suggest domain- and age-specific associations rather than a uniform or sustained pattern across development; confirmation in larger cohorts is needed.

## Key Points

**Question:**

Are cord serum concentrations of the neonicotinoid insecticide–related biomarkers dinotefuran and desmethyl-acetamiprid (dm-ACE) at birth associated with childhood neurodevelopment?

**Findings:**

This cohort study of 200 individuals in a Japanese birth cohort found associations of dinotefuran and dm-ACE with early childhood motor, visual reception, and receptive language scores; associations varied across age, and there were no associations after false discovery rate correction. Higher dinotefuran and dm-ACE concentrations were associated with lower processing speed and perceptual reasoning, respectively, at age 9 years.

**Meaning:**

These findings suggest that associations between neonicotinoid-related biomarkers and neurodevelopment may differ by assessment domain and between early and middle childhood.

## Teaser

This cohort study examines whether concentrations of neonicotinoid insecticide–related biomarkers in cord serum at birth are associated with neurodevelopment from infancy through middle childhood in a Japanese birth cohort.

## Introduction

The developing human brain is uniquely sensitive to environmental toxicants. During prenatal development, processes such as neurogenesis, neuronal migration, and synaptic organization establish the structural foundations of the brain.^1,2,3^ Disruption of these processes during gestation may have persistent implications for cognitive and behavioral functioning extending into later childhood.^4,5^ Because many environmental toxicants can cross the placenta,^6^ fetal brain development may be susceptible to maternal exposures even at relatively low levels.^7^ In this context of heightened vulnerability during prenatal brain development, neonicotinoid insecticides (NNIs) have emerged as a growing public health concern because of their widespread use and biological activity relevant to neurodevelopment.^8^

NNIs now account for more than one-quarter of global insecticide sales, driven by their low cost, high efficacy, and systemic properties.^8,9,10^ Their extensive use has resulted in widespread environmental contamination, with residues detected in food, drinking water, and indoor environments.^9,10,11,12^ In Japan, nationwide monitoring has demonstrated year-round detection of NNIs in tap water, suggesting that drinking water may represent a persistent exposure source.^13^ Correspondingly, human biomonitoring studies have consistently detected NNIs and their metabolites in biological samples from the general population, including pregnant individuals,^14,15,16^ reinforcing the relevance of prenatal exposure.

Experimental evidence provides biological plausibility for potential neurodevelopmental consequences of NNI exposure. NNIs act on nicotinic acetylcholine receptors (nAChRs), which have important roles in neuronal differentiation, synapse formation, and neural circuit maturation during fetal development.^17,18^ Disruption of nAChR-mediated signaling may therefore interfere with early brain development.^17,18^ Animal studies have reported altered brain morphology and impaired motor activity, learning, and attention-related behaviors following developmental NNI exposure, supporting further investigation of these findings in humans.^19,20,21^

Several observational studies have reported associations between prenatal NNI exposure and early neurodevelopmental outcomes; however, findings have been inconsistent.^22,23,24,25^ Previous studies generally assessed neurodevelopment at a single time point and did not extend beyond early childhood, limiting understanding of whether associations differ across developmental stages.^22,23,25,26^ Those studies also relied largely on maternal urinary biomarkers, which do not directly measure fetal exposure. Cord serum, in contrast, provides a direct measure of fetal exposure at delivery. Recent model-based pharmacokinetic evidence suggests longer human persistence of some parent NNIs, with estimated median biological half-lives of 53.7 days for dinotefuran and 15.5 days for acetamiprid.^27^ We therefore examined associations of cord serum concentrations of dinotefuran and desmethyl-acetamiprid (dm-ACE), a metabolite of the NNI acetamiprid, with domain-specific neurodevelopmental outcomes assessed from infancy through middle childhood in a Japanese birth cohort.

## Methods

### Study Design

This cohort study was conducted as part of an ongoing prospective birth cohort study, the Hamamatsu Birth Cohort for Mothers and Children (HBC Study). The HBC Study was initiated in 2007 to investigate early-life environmental, biological, and psychosocial factors associated with child development.^28^ Pregnant women were recruited during the first or second trimester of pregnancy at 2 research sites: the Hospital of Hamamatsu University School of Medicine and the Kato Maternity Clinic in Hamamatsu City, Japan. Women who delivered between December 2007 and March 2012 were eligible for participation; their children were subsequently followed up. The HBC Study design and procedures have been described elsewhere.^28,29^ All study procedures were approved by the ethics committee of Hamamatsu University School of Medicine and complied with the ethical principles of the Declaration of Helsinki.^30^ Written informed consent was obtained from all caregivers of the child participants. This study followed the Strengthening the Reporting of Observational Studies in Epidemiology (STROBE) reporting guideline.

### Participants

Among the individuals initially enrolled in the HBC Study, cord blood samples were collected at birth following standardized procedures (eMethods in Supplement 1). Samples with visible hemolysis were excluded from NNI measurement. Due to resource constraints, cord serum analyte concentrations were measured in a randomly selected subset of participants, who constituted the analytic sample (Figure 1). To assess potential selection bias, we compared baseline characteristics of the included participants with those of the participants not included in the analytic sample.

**Figure 1.**
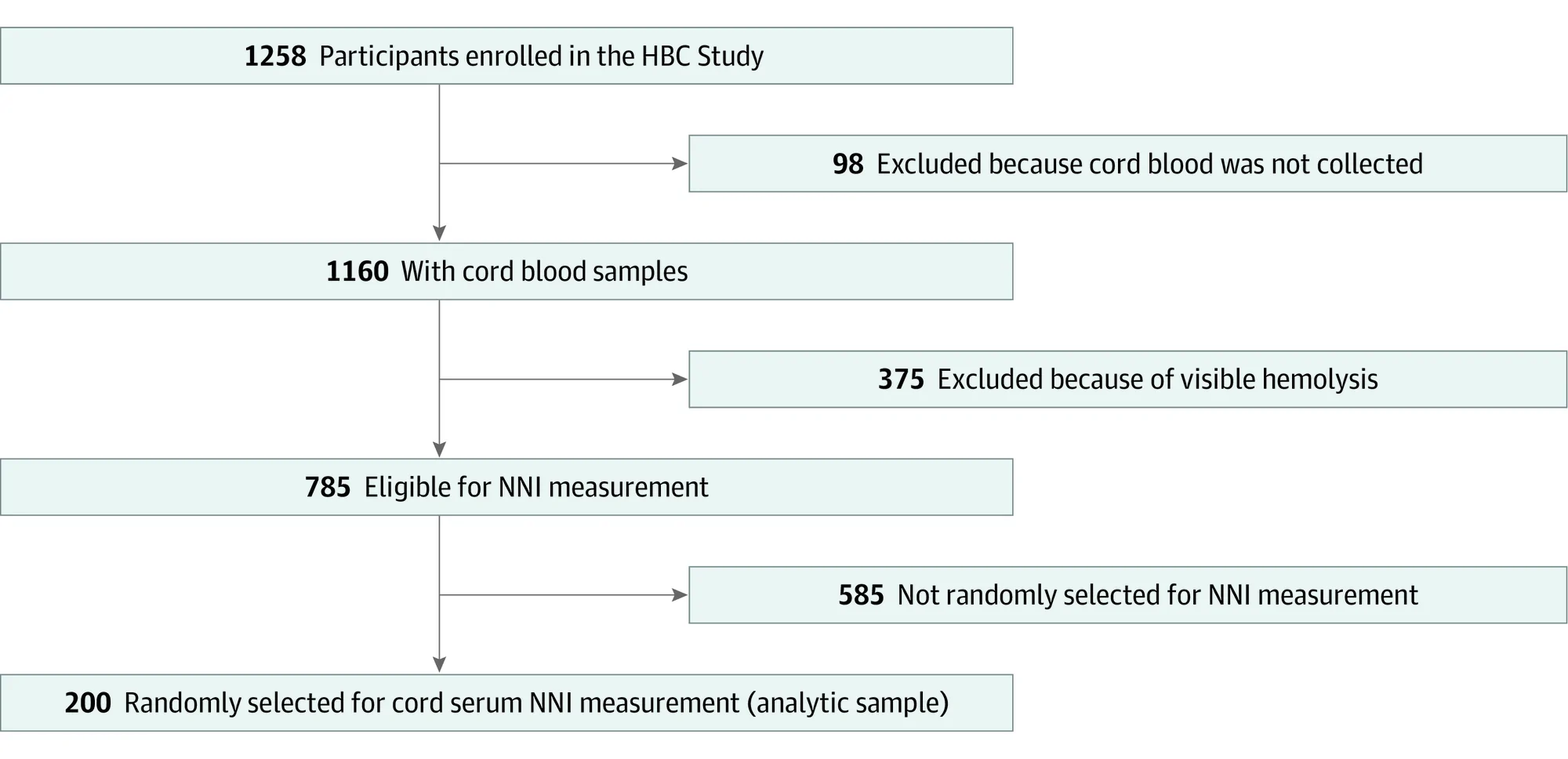
Flowchart of Participant Selection HBC indicates Hamamatsu Birth Cohort for Mothers and Children Study; NNI, neonicotinoid insecticide.

### Measurement

#### Neonicotinoid-Related Biomarkers

Neonicotinoid-related analyte concentrations (the exposures of interest) were measured in serum samples extracted from umbilical cord blood at birth. Ten targeted insecticides and their metabolites were quantified at Shimadzu Techno-Research, Inc using liquid chromatography–tandem mass spectrometry following solid-phase extraction (eMethods in Supplement 1). Compound-specific method detection limits (MDLs), detection frequencies, proportions at or above method quantification limits, and maximum concentrations are reported in eTable 1 in Supplement 1. Concentrations below the MDL were imputed as MDL divided by the square root of 2. Analytes detected in less than 30% of samples were excluded from inferential analyses because of insufficient variability.^25^ Dinotefuran and dm-ACE were the only analytes detected in at least 30% of samples and were therefore retained for analysis. Overall and insecticide and metabolite concentration group–specific distributions of dinotefuran and dm-ACE are presented in eTables 2 and 3 in Supplement 1.

#### Neurodevelopmental Outcomes

Early childhood neurodevelopment was assessed using the Mullen Scales of Early Learning (MSEL)^31^ at 5 prespecified HBC Study visits: ages 10, 14, 18, 24, and 32 months. The primary outcomes were 5 domain scores: gross motor, visual reception, fine motor, receptive language, and expressive language. Domain-specific scores were standardized using HBC Study reference distributions and expressed as *z* scores (mean, 0; SD, 1), with higher scores indicating more advanced neurodevelopment (details are provided in the eMethods in Supplement 1).^32^ To complement the domain-specific analyses, the official MSEL Early Learning Composite (ELC), calculated according to the MSEL manual from the 4 domains other than gross motor, was examined as a measure of overall neurodevelopment and retained in its original standard score units (normative mean, 100; SD, 15).

To assess cognitive functioning at a later developmental stage, children were evaluated at 9 years of age using the Japanese version of the Wechsler Intelligence Scale for Children, Fourth Edition (WISC-IV). The primary outcomes were the Verbal Comprehension Index, Perceptual Reasoning Index, Working Memory Index, and Processing Speed Index. To complement the index-specific analyses, full-scale IQ (FSIQ) was examined as a measure of overall cognitive ability. The 4 indices and FSIQ are norm-referenced scores (mean, 100; SD, 15) and were converted to *z* scores (mean, 0; SD, 1) for analysis, with higher scores indicating better cognitive functioning.^33^

#### Covariates

Covariates were selected based on previous literature, plausible associations with prenatal exposure or child neurodevelopment, and availability in the HBC Study.^22,23,25^ Given the modest sample size and the distribution of concentration groups, we used a parsimonious set of covariates to avoid model overfitting and unstable estimates. Primary models included exact child age at neurodevelopmental assessment, child sex, maternal and paternal educational attainment (assessed during pregnancy and used in all analyses as proxies for socioeconomic status), and preterm birth (<37 weeks’ gestation). Maternal smoking during pregnancy and alcohol use after 12 weeks’ gestation were included in sensitivity analyses.

### Statistical Analysis

Continuous variables were summarized as means (SDs), categorical variables as numbers and percentages, and dinotefuran and dm-ACE concentrations as medians and IQRs. Characteristics of included and excluded participants were compared using 2-sided *t* tests or Pearson χ^2^ tests, as appropriate. Because the substantial proportions of concentrations below the MDLs were assigned identical imputed values, quartile 1 (Q1) and Q2 could not be meaningfully distinguished. Concentrations were therefore categorized as Q1 to Q2 (reference), Q3, and Q4. Identical concentrations at empirical category boundaries were retained in the same category rather than split arbitrarily, resulting in unequal group sizes.

Associations of cord serum dinotefuran and dm-ACE concentrations with early childhood MSEL domain scores were examined separately at each assessment age using generalized structural equation modeling. The 5 domain *z* scores were modeled simultaneously in separate linear equations using a common complete-case sample. Crude models included each biomarker separately, whereas adjusted models included both biomarkers and the aforementioned covariates. Associations with the ELC at each age and with the WISC-IV indices and FSIQ at age 9 years were examined using linear regression. Estimates were expressed as mean differences with 95% CIs comparing Q3 and Q4 with Q1 to Q2.

In additional analyses, potential concentration-response associations were evaluated using linear trend models that assigned each concentration group its median log_2_ concentration and continuous models based on individual log_2_-transformed concentrations. Continuous-model estimates represented mean differences per doubling of concentration. Longitudinal associations across repeated MSEL assessments were examined separately for each domain using gaussian identity-link generalized estimating equations (GEEs) with an exchangeable working correlation structure. Models without concentration × age interactions estimated population-averaged associations across ages, whereas interaction models provided omnibus concentration × age tests and age-specific contrasts.

To address multiple comparisons, the Benjamini-Hochberg procedure was applied separately to dinotefuran and dm-ACE within 3 families of adjusted analyses: MSEL domains (50 tests per biomarker: 5 domains ×  5 ages × 2 contrasts), WISC-IV indices and FSIQ (10 tests per biomarker: 5 outcomes × 2 contrasts), and ELC (10 tests per biomarker: 5 ages × 2 contrasts). Unadjusted *P* values and false discovery rate (FDR)–adjusted *q* values are reported.

In sensitivity analyses, the adjusted models for the MSEL domains, WISC-IV indices, and FSIQ were repeated with additional adjustment for maternal smoking and alcohol use. Separate models omitted preterm birth because it may lie partly on the pathway between NNI-related biomarker concentrations and neurodevelopment. Outcome-specific samples were held constant across the corresponding primary and sensitivity models. Maternal cluster-robust SEs were used throughout. Analyses used complete case data. All tests were 2 sided, with *P* < .05 considered nominally significant and *q* < .05 considered significant after FDR correction. Analyses were conducted using Stata, version 18 (StataCorp LLC) from October 2025 to September 2026.

## Results

### Participant Characteristics and Biomarker Distributions

Of the 1258 children in the HBC Study, cord blood samples were collected at birth from 1160. Of these samples, 375 were excluded due to visible hemolysis, leaving 785 eligible for NNI measurement. Cord serum analyte concentrations were measured in a randomly selected subset of 200 participants in the analytic sample, of whom 98 (49.0%) were female, 102 (51.0%) were male, and 13 (6.5%) were born preterm (Table). Age-specific complete case samples for the primary MSEL models ranged from 185 (92.5%) to 195 (97.5%) of the participants, and 178 participants (89.0%) were included in the WISC-IV analyses at age 9 years. Characteristics of the 200 included and 1058 excluded participants were generally similar, although the mean (SD) paternal educational level was higher among included participants (14.6 [2.6] vs 14.0 [2.7] years; *P* = .008).

**Table.** Participant Characteristics and Neurodevelopmental Outcomes

Characteristic	Participants^a^			*P* value^b^
	Final sample (n = 200)		Excluded cohort (n = 1058)	
	No.	Value		
Parent educational level, y				
Maternal	200	13.8 (1.8)	13.8 (2.0)	.94
Paternal	200	14.6 (2.6)	14.0 (2.7)	.008
Sex, No. (%)				
Female	200	98 (49.0)	510 (48.2)	.84
Male	200	102 (51.0)	548 (51.8)	
Preterm birth (<37 wk gestation), No. (%)				
No	200	187 (93.5)	985 (93.1)	.84
Yes	200	13 (6.5)	73 (6.9)	
Maternal smoking during pregnancy, No. (%)				
No	200	188 (94.0)	966 (91.3)	.20
Yes	200	12 (6.0)	92 (8.7)	
Maternal alcohol use after 12 wk gestation, No. (%)				
No	200	172 (86.0)	927 (87.6)	.53
Yes	200	28 (14.0)	131 (12.4)	
MSEL score, mean (SD)				
10 mo				
T score				
Gross motor	198	47.8 (8.9)	NA	NA
Visual reception	198	47.7 (10.8)	NA	NA
Fine motor	197	48.1 (10.2)	NA	NA
Receptive language	196	46.2 (10.1)	NA	NA
Expressive language	197	46.8 (8.7)	NA	NA
ELC	195	96.2 (15.1)	NA	NA
14 mo				
T score				
Gross motor	195	48.4 (10.5)	NA	NA
Visual reception	197	48.1 (9.8)	NA	NA
Fine motor	197	49.5 (10.0)	NA	NA
Receptive language	190	48.3 (10.3)	NA	NA
Expressive language	196	48.8 (11.0)	NA	NA
ELC	188	97.6 (13.9)	NA	NA
18 mo				
T score				
Gross motor	196	48.4 (9.1)	NA	NA
Visual reception	199	48.0 (9.6)	NA	NA
Fine motor	200	49.3 (10.1)	NA	NA
Receptive language	194	48.2 (10.5)	NA	NA
Expressive language	197	48.6 (10.0)	NA	NA
ELC	191	97.5 (15.9)	NA	NA
24 mo				
T score				
Gross motor	196	48.4 (10.6)	NA	NA
Visual reception	200	48.5 (10.0)	NA	NA
Fine motor	198	48.3 (9.8)	NA	NA
Receptive language	192	50.3 (9.5)	NA	NA
Expressive language	195	49.6 (9.4)	NA	NA
ELC	189	98.7 (12.5)	NA	NA
32 mo				
T score				
Gross motor	189	49.4 (10.4)	NA	NA
Visual reception	194	49.1 (9.3)	NA	NA
Fine motor	196	50.1 (10.0)	NA	NA
Receptive language	196	49.9 (9.1)	NA	NA
Expressive language	195	49.3 (10.5)	NA	NA
ELC	192	99.6 (14.6)	NA	NA
WISC-IV at age 9 y				
Verbal Comprehension Index	178	105.5 (13.3)	NA	NA
Perceptual Reasoning Index	178	100.4 (15.0)	NA	NA
Working Memory Index	178	97.9 (14.5)	NA	NA
Processing Speed Index	178	99.7 (13.5)	NA	NA
Full-scale IQ	178	102.2 (13.7)	NA	NA

Abbreviations: ELC, Early Learning Composite; MSEL, Mullen Scales of Early Learning; NA, not applicable; WISC-IV, Wechsler Intelligence Scale for Children, Fourth Edition.

^a^ Data are presented as mean (SD) values unless otherwise indicated.

^b^ Two-sided *t* test or Pearson χ^2^ test, as appropriate.

Dinotefuran and dm-ACE were detected in 104 (52.0%) and 125 (62.5%) of the analyte samples, respectively, compared with 0 to 18 (9.0%) for all other analytes (eTable 1 in Supplement 1). For dinotefuran, the Q1 and Q2 group included 96 cases (48.0%), with median concentration of 0.02 ng/mL (IQR, 0.02-0.02 ng/mL), whereas the Q3 group included 47 cases (23.5%) with median concentration of 0.04 ng/mL (IQR, 0.04-0.05 ng/mL) and the Q4 group included 57 cases (28.5%) with median concentration of 0.07 ng/mL (IQR, 0.06-0.08 ng/mL). The corresponding dm-ACE groups included 82 cases (41.0%) in Q1 and Q2 with median concentration of 0.01 ng/mL (IQR, 0.01-0.01 ng/mL); 63 (31.5%) in Q3, with median concentration of 0.04 ng/mL (IQR, 0.03-0.06 ng/mL); and 55 (27.5%) in Q4, with median concentration of 0.11 ng/mL (IQR, 0.10-0.18 ng/mL) (eTables 1-3 in Supplement 1).

### Associations With Early Childhood Neurodevelopment

In adjusted age-specific analyses, the Q3 dinotefuran concentration compared with Q1 and Q2 was associated with lower gross motor scores at ages 10 months (mean difference, −0.36 SDs; 95% CI, −0.65 to −0.06 SDs) and 14 months (mean difference, −0.41 SDs; 95% CI, −0.78 to −0.03 SDs), and Q4 dinotefuran was associated with lower fine motor scores at age 24 months (mean difference, −0.35 SDs; 95% CI, −0.65 to −0.05 SDs) (Figure 2). Q3 dinotefuran concentration was associated with lower receptive language scores at age 14 months (mean difference, −0.39 SDs; 95% CI, −0.73 to −0.05 SDs) (Figure 2).

**Figure 2.**
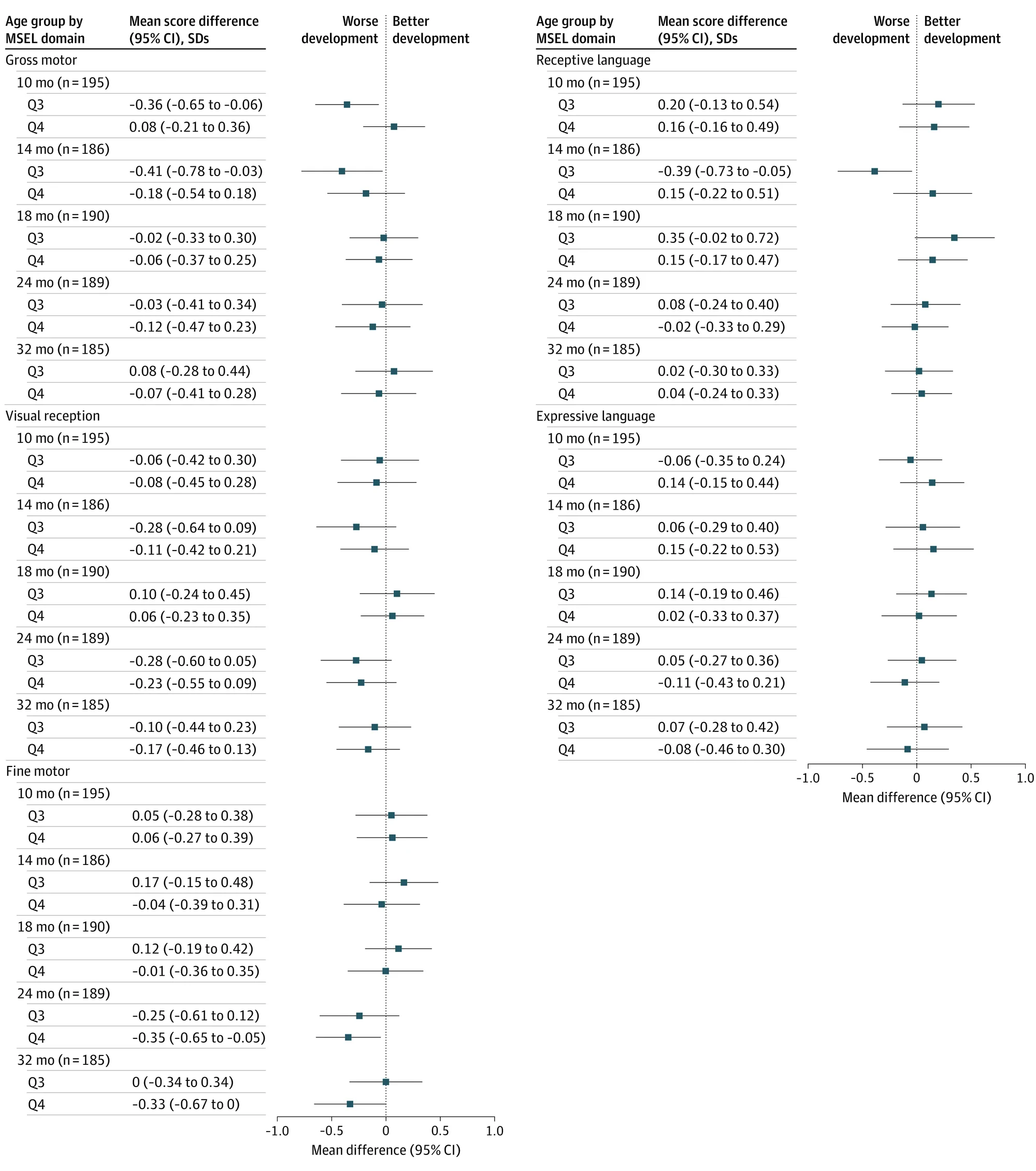
Dot-and-Whisker Plots Showing Associations of Dinotefuran Concentration Quartiles With Differences in Mullen Scales of Early Learning (MSEL) Domain Scores at Ages 10-32 Months Concentration groups were based on quartiles of dinotefuran concentrations measured in umbilical cord serum (highest was quartile 4 [Q4]). At each assessment age, the 5 MSEL domain equations were estimated simultaneously using a common complete-case sample. Higher MSEL scores indicate more advanced neurodevelopment. Models were adjusted for exact age at assessment, child sex, maternal and paternal educational levels, and preterm birth. SEs were clustered at the maternal level. Markers indicate adjusted mean differences in standardized MSEL scores for Q3 and Q4 compared with combined Q1-Q2 as the reference, and whiskers indicate 95% CIs. Mean differences are expressed in SD units.

For dm-ACE, Q4 concentration was associated with lower visual reception scores (mean difference, −0.36 SDs; 95% CI, −0.69 to −0.03 SDs) and lower fine motor scores (−0.46 SDs; 95% CI, −0.79 to −0.14 SDs) at age 10 months (Figure 3). In contrast, Q3 dm-ACE concentration was associated with higher fine motor scores at age 18 months (mean difference, 0.32 SDs; 95% CI, 0.01-0.64 SDs) (Figure 3). None of these associations remained statistically significant after FDR correction (eTables 4 and 5 in Supplement 1). No consistent concentration group pattern was evident across ages or MSEL domains.

**Figure 3.**
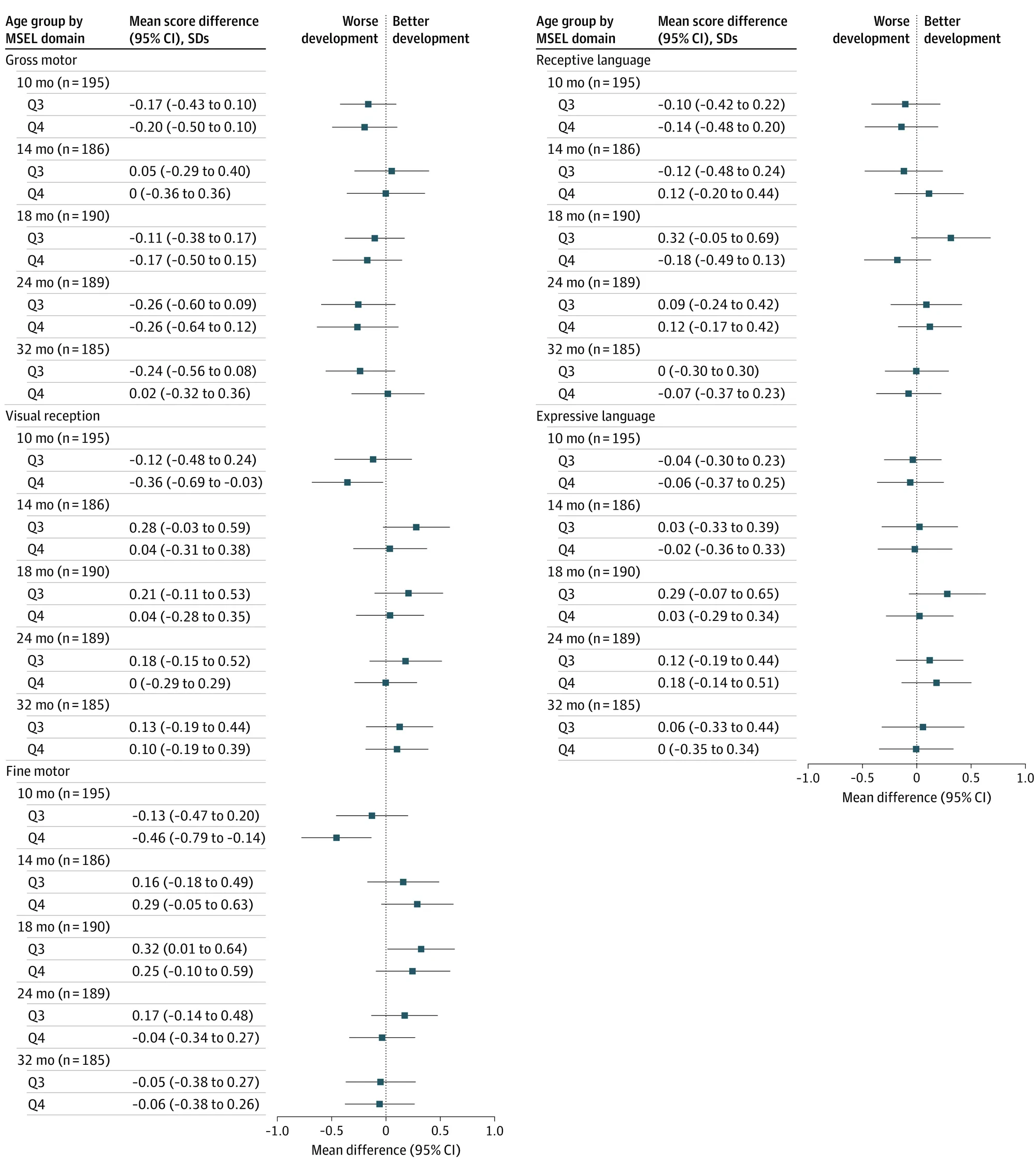
Dot-and-Whisker Plots Showing Associations of Desmethyl-Acetamiprid (dm-ACE) Concentration Quartiles With Mullen Scales of Early Learning (MSEL) Domain Scores at Ages 10-32 Months Concentration groups were based on quartiles of dm-ACE concentrations measured in umbilical cord serum (highest was quartile 4 [Q4]). At each assessment age, the 5 MSEL domain equations were estimated simultaneously using a common complete-case sample. Higher MSEL scores indicate more advanced neurodevelopment. Models were adjusted for exact age at assessment, child sex, maternal and paternal educational levels, and preterm birth. SEs were clustered at the maternal level. Markers indicate adjusted mean differences in standardized MSEL scores for Q3 and Q4 compared with combined Q1-Q2 as the reference, and whiskers indicate 95% CIs. Mean differences are expressed in SD units.

In supplementary repeated-measures GEE analyses, neither dinotefuran nor dm-ACE concentration was associated with population-averaged MSEL domain scores across assessments. Nominal concentration × age interactions were observed for dinotefuran in gross motor and receptive language scores and for dm-ACE in fine motor and receptive language scores. However, the age-specific estimates varied in direction and did not demonstrate consistent concentration group patterns (eTable 6 in Supplement 1). The MSEL estimates remained consistent with the primary estimates after additional adjustment for maternal smoking and alcohol use during pregnancy (eTable 7 in Supplement 1).

### Associations With Middle Childhood Cognitive Function

Among 178 children (89.0%) assessed at age 9 years, Q3 dinotefuran concentration was associated with lower WISC-IV Processing Speed Index scores (mean difference, −0.50 SDs; 95% CI, −0.81 to −0.20 SDs; *P* = .001; *q* = .01) (Figure 4). Q4 dm-ACE concentration was associated with lower Perceptual Reasoning Index scores (mean difference, −0.47 SDs; 95% CI, −0.81 to −0.14 SDs; *P* = .006; *q* = .03) (Figure 4 and eTable 8 in Supplement 1). In complementary FSIQ analyses, lower scores were observed for Q3 dinotefuran concentration (mean difference, −0.46 SDs; 95% CI, −0.78 to −0.15 SDs; *P* = .005; *q* = .02) and Q4 dm-ACE concentration (mean difference, −0.46 SDs; 95% CI, −0.74 to −0.17 SDs; *P* = .002; *q* = .02). There were no other associations with WISC-IV indices after FDR correction.

**Figure 4.**
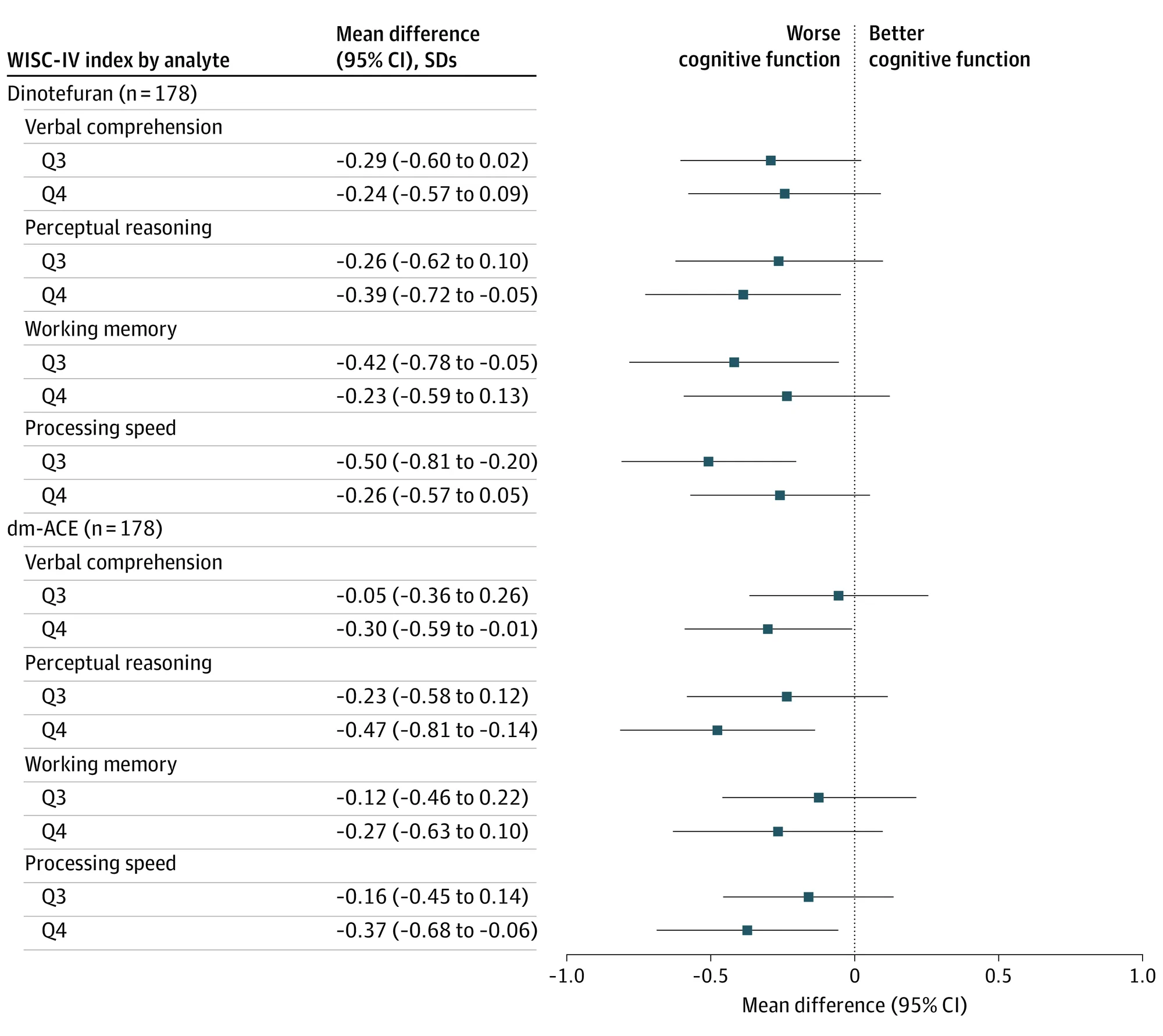
Dot-and-Whisker Plots Showing Associations of Dinotefuran and Desmethyl-Acetamiprid (dm-ACE) Concentration Groups With Wechsler Intelligence Scale for Children, Fourth Edition (WISC-IV) Index Scores at Age 9 Years Concentration groups were based on quartiles of dinotefuran and dm-ACE concentrations measured in umbilical cord serum (highest was quartile 4 [Q4]). Multivariable linear regression models included dinotefuran and dm-ACE simultaneously and were adjusted for exact age at assessment, child sex, maternal and paternal educational levels, and preterm birth. SEs were clustered at the maternal level. Markers indicate adjusted mean differences for Q3 and Q4 compared with combined Q1-Q2 as the reference, and whiskers indicate 95% CIs. Mean differences are expressed in SD units. Negative and positive estimates indicate lower and higher standardized WISC-IV index scores, respectively, compared with the Q1-Q2 reference.

The WISC-IV and FSIQ estimates remained consistent with those from the main analyses after additional adjustment for maternal smoking and alcohol use during pregnancy (eTable 9 in Supplement 1). Estimates for the MSEL, WISC-IV, and FSIQ outcomes also remained consistent when preterm birth was omitted from the covariate set (eTable 10 in Supplement 1).

### Complementary and Additional Analyses

Complementary composite and concentration-response analyses did not materially alter the interpretation of the primary findings. Detailed results and comparisons according to WISC-IV data availability are provided in the eResults and eTables 8 and 11 to 14 in Supplement 1.

## Discussion

In this prospective birth cohort study, the primary age-specific analyses identified associations of cord serum dinotefuran concentration with lower gross motor scores at ages 10 and 14 months, lower receptive language scores at age 14 months, and lower fine motor scores at age 24 months. Cord serum dm-ACE concentration was associated with lower fine motor and visual reception scores at age 10 months and higher fine motor scores at age 18 months. However, none of these early childhood associations were found after FDR correction. At age 9 years, dinotefuran was associated with lower processing speed and dm-ACE with lower perceptual reasoning after FDR correction. Overall, associations with select aspects of cognitive function were observed in middle childhood, but the findings regarding early childhood were less consistent.

Although associations varied across ages and concentration groups, most inverse early childhood estimates involved motor and visual-perceptual domains. Motor development relies on the coordinated maturation of cerebellar, cortical, and subcortical networks,^34^ which develop during late gestation and early postnatal life. Biological plausibility for the association of prenatal dinotefuran and dm-ACE exposure with motor development is further supported by the role of nAChRs in neuronal differentiation, synaptic organization, and sensorimotor processing and by experimental evidence that NNIs can alter cholinergic signaling in developing neural circuits.^35,36^ Visual reception similarly involves developing visuospatial and attentional systems.^31,37^ These neural systems may therefore be vulnerable to environmental perturbations during sensitive developmental periods. Wang et al^25^ previously reported inverse associations between prenatal NNI exposure and early psychomotor and cognitive development by 2 years of age. However, direct comparison with previous observational studies is challenging because of the limited number of studies and differences in exposure assessment and neurodevelopmental outcomes. Nevertheless, variation in associations across ages and NNI concentration groups argues against a uniform adverse early childhood pattern.

This study’s findings regarding school-aged children may have some conceptual correspondence with those regarding early childhood. The clearest example was the association of Q4 dm-ACE concentration with lower visual reception at age 10 months and lower perceptual reasoning at age 9 years. Visual reception tasks in the MSEL assess early visuospatial perception, pattern recognition, and attention, which provide foundations for later visuospatial reasoning and cognitive performance.^31,37^ During infancy, visual cortical pathways and frontoparietal attention networks undergo rapid maturation, supporting visual attention and perceptual integration.^31,33,38^ Developmental research has also shown an association of early visual perceptual and motor abilities with later cognitive development.^34,39,40,41,42^ The occurrence of conceptually related findings at 2 developmental stages in this study may therefore be noteworthy. However, the present analyses did not test whether early MSEL differences mediated WISC-IV performance later. Accordingly, this correspondence may suggest continuity between early perceptual differences and later cognitive performance, but the present analyses could not establish such continuity.

The complementary analyses in this study support a domain-specific interpretation: ELC findings provided little evidence of generalized differences in early learning performance, whereas FSIQ findings were consistent with broader cognitive differences at school age. Population-averaged GEE estimates may have been attenuated because age-specific associations that varied in direction were averaged together. Nevertheless, the inconsistent concentration × age and dose-response findings do not support a sustained developmental pattern. Given the modest sample size, the absence of associations for other domains does not exclude smaller differences.

### Strengths and Limitations

Strengths of this study included use of cord serum biomarkers of fetal exposure to NNIs, repeated standardized MSEL assessments followed by WISC-IV assessment in middle childhood, mutually adjusted biomarker models, and multiple-comparison correction. Several limitations should also be considered. First, the modest sample size limited statistical precision, particularly within concentration groups, and our ability to detect smaller associations or examine effect size modification. Multiple comparisons increased the possibility of chance findings, although FDR correction was applied to the primary and complementary adjusted analyses. Second, despite random selection, paternal educational attainment was higher among included than excluded participants, and dinotefuran concentrations were higher among children with complete WISC-IV data than those without; thus, selection bias cannot be excluded. Because participants came from a single Japanese birth cohort, generalizability to populations with different demographic or exposure characteristics may be limited.

Third, a single cord serum measurement reflects fetal exposure at delivery but cannot characterize the timing or variability of exposure throughout pregnancy. Although pharmacokinetic evidence suggests that dinotefuran may persist longer in humans than was previously recognized,^27^ uncertainty remains regarding how well a single measurement represents exposure across gestation. Concentration categorization and imputation below the MDL may also have introduced measurement error. Fourth, low detection frequencies for other analytes precluded assessment of their individual or combined neurodevelopmental associations. Fifth, parental cognitive functioning, dietary patterns, and developmental neurotoxicants such as lead and other heavy metals were not measured, and residual confounding cannot be excluded. Parental educational level may not fully capture parental cognitive functioning, and correlations between environmental coexposures and dinotefuran or dm-ACE could not be assessed. Sixth, because only 13 children were born preterm, potential mediation through preterm birth could not be formally evaluated.

## Conclusions

In this prospective Japanese birth cohort study, associations of cord serum concentrations of the neonicotinoid-related biomarkers dinotefuran and dm-ACE with motor, visual reception, and receptive language scores varied across early childhood assessments. At age 9 years, lower processing speed was associated with dinotefuran and lower perceptual reasoning was associated with dm-ACE. Although variation across ages and domains argues against a uniform or sustained pattern of association, null findings cannot exclude smaller or age-specific associations. These findings warrant confirmation in larger cohorts with repeated measurements of prenatal exposure and longitudinal neurodevelopmental follow-up through middle childhood.

## Notes

Supplement 1. **eMethods.** Supplementary Methods **eResults.** Supplementary Results **eTable 1.** Detection Frequencies and Concentrations of Target Insecticides and Metabolites in Cord Serum (n = 200) **eTable 2.** Distribution of Cord Serum DIN and dm-ACE Concentrations (n = 200) **eTable 3.** Cord Serum DIN and dm-ACE Concentrations by Group (n = 200) **eTable 4.** Associations of Cord Serum DIN Concentration Groups With MSEL Domain Scores **eTable 5.** Associations of Cord Serum dm-ACE Concentration Groups With MSEL Domain Scores **eTable 6.** Overall and Age-Specific GEE Associations of Cord Serum DIN and dm-ACE With MSEL Domain Scores **eTable 7.** Associations of Cord Serum DIN and dm-ACE With Age-Specific Standardized MSEL Domain Scores After Additional Adjustment for Maternal Smoking and Alcohol Use During Pregnancy **eTable 8.** Associations of Cord Serum DIN and dm-ACE With WISC-IV Indices and Full-Scale IQ at 9 Years of Age **eTable 9.** Associations of Cord Serum DIN and dm-ACE With WISC-IV Indices and Full-Scale IQ After Additional Adjustment for Maternal Smoking and Alcohol Use During Pregnancy **eTable 10.** Sensitivity Analyses of Associations of Cord Serum DIN and dm-ACE With MSEL and WISC-IV Outcomes Without Adjustment for Preterm Birth **eTable 11.** Associations of Cord Serum DIN and dm-ACE With MSEL Early Learning Composite Scores at 10 to 32 Months **eTable 12.** Trend and Continuous-Concentration Associations of Cord Serum DIN and dm-ACE With Age-Specific Standardized MSEL Domain Scores **eTable 13.** Trend and Continuous-Concentration Associations of Cord Serum DIN and dm-ACE With WISC-IV Indices and Full-Scale IQ at Age 9 Years **eTable 14.** Characteristics of Participants With and Without Complete WISC-IV Data at Age 9 Years
eReference

Supplement 2.
Data Sharing Statement
