# Supplementary material for: Cord Serum Neonicotinoid-Related Biomarkers and Neurodevelopment From Early to Middle Childhood
Source: JAMA Netw Open. 2026 Nov 11;9(11):e2643481. doi: 10.1001/jamanetworkopen.2026.43481 (PMC13756127; doi:10.1001/jamanetworkopen.2026.43481)
Supplement: Supplementary file 1 [file jamanetwopen-e2643481-s001.pdf]

Supplementary Online Content

Nagata A, Okumura A, Takahashi N, et al. Cord serum neonicotinoid-related biomarkers and neurodevelopment from early to middle childhood. *JAMA Netw Open*. 2026;9(11):e2643481. doi:10.1001/jamanetworkopen.2026.43481

eMethods. Supplementary Methods

eResults. Supplementary Results

eTable 1. Detection Frequencies and Concentrations of Target Insecticides and Metabolites in Cord Serum (n=200)

eTable 2. Distribution of Cord Serum DIN and dm-ACE Concentrations (n=200)

eTable 3. Cord Serum DIN and dm-ACE Concentrations by Group (n=200)

eTable 4. Associations of Cord Serum DIN Concentration Groups With MSEL Domain Scores

eTable 5. Associations of Cord Serum dm-ACE Concentration Groups With MSEL Domain Scores

eTable 6. Overall and Age-Specific GEE Associations of Cord Serum DIN and dm-ACE With MSEL Domain Scores

eTable 7. Associations of Cord Serum DIN and dm-ACE With Age-Specific Standardized MSEL Domain Scores After Additional Adjustment for Maternal Smoking and Alcohol Use During Pregnancy

eTable 8. Associations of Cord Serum DIN and dm-ACE With WISC-IV Indices and Full-Scale IQ at 9 Years of Age

eTable 9. Associations of Cord Serum DIN and dm-ACE With WISC-IV Indices and Full-Scale IQ After Additional Adjustment for Maternal Smoking and Alcohol Use During Pregnancy

eTable 10. Sensitivity Analyses of Associations of Cord Serum DIN and dm-ACE With MSEL and WISC-IV Outcomes Without Adjustment for Preterm Birth

eTable 11. Associations of Cord Serum DIN and dm-ACE With MSEL Early Learning Composite Scores at 10 to 32 Months

eTable 12. Trend and Continuous-Concentration Associations of Cord Serum DIN and dm-ACE With Age-Specific Standardized MSEL Domain Scores

eTable 13. Trend and Continuous-Concentration Associations of Cord Serum DIN and dm-ACE With WISC-IV Indices and Full-Scale IQ at Age 9 Years

© 2026 Nagata A et al. *JAMA Network Open*.

eTable 14. Characteristics of Participants With and Without Complete WISC-IV Data at Age 9 Years

eReference

This supplementary material has been provided by the authors to give readers additional information about their work.

© 2026 Nagata A et al. *JAMA Network Open*.

eMethods

Cord blood collection and sample preparation

Umbilical cord blood samples were collected immediately after delivery from the umbilical vein. Samples were maintained at room temperature for approximately 30 minutes and subsequently centrifuged at 3,500 revolutions per minute for 10 minutes to separate serum. The serum fraction was aliquoted into polypropylene tubes and stored at −80 °C until analysis. For chemical analysis, 0.5 mL of cord serum was spiked with 2 ng of internal standard, followed by the addition of 1 mL of methanol to precipitate proteins. After protein removal, 10 mL of water and 0.1 mL of formic acid were added to the supernatant. The resulting solution was loaded onto a solid-phase extraction cartridge (Oasis WAX). Target analytes were eluted with 5 mL of methanol containing 0.1% ammonia. The eluate was concentrated under a gentle stream of nitrogen gas to a volume of less than 0.5 mL, and the final measurement solution was adjusted to 0.5 mL prior to instrumental analysis.

Neonicotinoid Insecticides and Metabolites

Determination of Detection and Quantification Limits

Instrumental detection limits were estimated from replicate measurements of low-concentration standard solutions. Method detection limits (MDLs) and method quantification limits (MQLs) were estimated from replicate analyses of fortified blank samples processed using the same procedures as study samples. Calculations followed established analytical chemistry guidelines. Compound-specific MDLs and detection frequencies, together with the proportions of samples with concentrations at or above the MQL, are reported in eTable 1.

Quality Control

Analytical quality-control procedures included internal standard correction, calibration curves evaluated for linearity, and replicate measurements to monitor analytical precision. Samples were processed and analyzed according to the same protocol across analytical batches.

Neurodevelopmental Outcomes

Early Childhood Assessment

At each early-childhood follow-up, the MSEL was administered face-to-face in a dedicated participant-assessment room at the Research Center for Child Mental Development, Hamamatsu University School of Medicine, with a parent present. A team of approximately 10 trained project assistants or project researchers conducted the assessments across the follow-up waves, with at least 2 project staff members present during each visit. Examiners completed up to 3 months of interactive training using video-recorded child-development assessments and participated in refresher and quality-maintenance sessions before each follow-up wave to promote consistent administration and scoring. The closely spaced visits (10, 14, 18, 24, and 32 months) were prespecified in the HBC Study to characterize rapid developmental change during infancy. Because MSEL scores were age-referenced, assessments were analyzed separately by age; repeated-measures GEE analyses were used as a complementary approach.[1] Raw MSEL domain scores were converted to T scores using HBC Study–derived reference distributions and subsequently standardized as z scores for analysis. The ELC was calculated according to the MSEL manual and retained in its original standard-score units.[1]

Middle Childhood Assessment

At the age-9 follow-up, the Japanese version of the WISC-IV was administered face-to-face in a dedicated participant-assessment room at the same research center. Children completed the assessment

© 2026 Nagata A et al. *JAMA Network Open*.

without a parent present, while their parents remained in a separate room. A team of approximately 7 trained project assistants or project researchers administered the WISC-IV during the age-9 follow-up using standardized administration and scoring procedures.

© 2026 Nagata A et al. *JAMA Network Open*.

eResults

Complementary and Additional Analyses

Complementary FSIQ analyses showed lower scores for DIN Q3 and dm-ACE Q4, both of which remained significant after FDR correction (eTable 8). Complementary ELC analyses showed dm-ACE associations at 10 and 18 months; neither remained significant after FDR correction (eTable 11). Trend and continuous-concentration analyses showed no consistent pattern across MSEL ages or domains (eTable 12). At age 9 years, both approaches yielded nominal inverse associations of DIN and dm-ACE with PRI and FSIQ, whereas findings for PSI were less consistent (eTable 13).

WISC-IV Data Availability

Among the 200 children, 178 (89.0%) had complete WISC-IV data. Except for DIN, measured characteristics differed only slightly according to WISC-IV data availability (absolute standardized differences, 0.03–0.13). Median DIN concentrations were higher among children with complete WISC-IV data than those without (0.040 vs 0.021 ng/mL; standardized difference, 0.33) (eTable 14).

© 2026 Nagata A et al. *JAMA Network Open*.

eTable 1. Detection Frequencies and Concentrations of Target Insecticides and Metabolites in Cord Serum (n=200)

**Analyte**	**Detection frequency (%)**	**MDL (ng/mL)**	**Frequency ≥ MQL (%)**	**Maximum (ng/mL)**
Clothianidin (CLO)	1	0.01	1	0.09
Acetamiprid (ACE)	6	0.03	4	0.18
Imidacloprid (IMI)	9	0.04	2	0.18
Thiacloprid (THI)	1	0.03	0	NA
Thiamethoxam (THIA)	2	0.02	0	NA
Dinotefuran (DIN)	52.0	0.03	5	0.26
Nitenpyram (NIT)	0	0.02	0	NA
Fipronil (FIP)	0	0.01	0	NA
Desmethyl-acetamiprid (dm-ACE)	62.5	0.02	41	1.3
Desmethyl Thiamethoxam (dm-THIA)	0	0.10	0	NA

Abbreviations: MDL, method detection limit; MQL, method quantification limit; NA, not applicable
Maximum concentrations are reported only for analytes with at least 1 sample at or above the MQL; NA indicates that no sample met the MQL.

© 2026 Nagata A et al. *JAMA Network Open*.

eTable 2. Distribution of Cord Serum DIN and dm-ACE Concentrations (n=200)

**Percentile**	**DIN (ng/mL)**	**dm-ACE (ng/mL)**
25th	0.02	0.01
50th	0.03	0.03
75th	0.06	0.08
95th	0.08	0.20
Maximum	0.26	1.30

Abbreviations: DIN, dinotefuran; dm-ACE, desmethyl-acetamiprid

© 2026 Nagata A et al. *JAMA Network Open*.

eTable 3. Cord Serum DIN and dm-ACE Concentrations by Group (n=200)

Group	No. (%)	Median (ng/mL)	IQR (ng/mL)
**DIN**			
Q1–Q2 (reference)	96 (48.0)	0.02	0.02 – 0.02
Q3	47 (23.5)	0.04	0.04 – 0.05
Q4	57 (28.5)	0.07	0.06 – 0.08
**dm-ACE**			
Q1–Q2 (reference)	82 (41.0)	0.01	0.01 – 0.01
Q3	63 (31.5)	0.04	0.03 – 0.06
Q4	55 (27.5)	0.11	0.10 – 0.18

Abbreviations: DIN, dinotefuran; dm-ACE, desmethyl-acetamiprid; IQR, interquartile range

© 2026 Nagata A et al. *JAMA Network Open*.

eTable 4. Associations of Cord Serum DIN Concentration Groups With MSEL Domain Scores

Age, mo	MSEL domain	Comparison	Participants, No.	Crude mean difference (95% CI)	*P* value	Adjusted mean difference (95% CI)[a]	*P* value	FDR *q* value[b]
10	Gross motor	Q3 vs Q1-Q2	195	-0.23 (-0.56, 0.09)	.156	-0.36 (-0.65, -0.06)	.017	.423
10	Gross motor	Q4 vs Q1-Q2	195	0.23 (-0.05, 0.51)	.108	0.08 (-0.21, 0.36)	.599	.915
10	Visual reception	Q3 vs Q1-Q2	195	0.04 (-0.35, 0.43)	.842	-0.06 (-0.42, 0.30)	.755	.915
10	Visual reception	Q4 vs Q1-Q2	195	0.09 (-0.26, 0.44)	.602	-0.08 (-0.45, 0.28)	.653	.915
10	Fine motor	Q3 vs Q1-Q2	195	0.14 (-0.21, 0.50)	.428	0.05 (-0.28, 0.38)	.767	.915
10	Fine motor	Q4 vs Q1-Q2	195	0.12 (-0.20, 0.45)	.454	0.06 (-0.27, 0.39)	.729	.915
10	Receptive language	Q3 vs Q1-Q2	195	0.33 (-0.00, 0.66)	.051	0.20 (-0.13, 0.54)	.233	.915
10	Receptive language	Q4 vs Q1-Q2	195	0.22 (-0.11, 0.55)	.189	0.16 (-0.16, 0.49)	.333	.915
10	Expressive language	Q3 vs Q1-Q2	195	0.04 (-0.25, 0.34)	.780	-0.06 (-0.35, 0.24)	.694	.915
10	Expressive language	Q4 vs Q1-Q2	195	0.21 (-0.08, 0.49)	.151	0.14 (-0.15, 0.44)	.345	.915
14	Gross motor	Q3 vs Q1-Q2	186	-0.39 (-0.79, 0.01)	.057	-0.41 (-0.78, -0.03)	.034	.423
14	Gross motor	Q4 vs Q1-Q2	186	-0.12 (-0.44, 0.20)	.479	-0.18 (-0.54, 0.18)	.319	.915
14	Visual reception	Q3 vs Q1-Q2	186	-0.26 (-0.64, 0.12)	.181	-0.28 (-0.64, 0.09)	.141	.884
14	Visual reception	Q4 vs Q1-Q2	186	-0.01 (-0.34, 0.31)	.943	-0.11 (-0.42, 0.21)	.514	.915
14	Fine motor	Q3 vs Q1-Q2	186	0.16 (-0.17, 0.49)	.335	0.17 (-0.15, 0.48)	.306	.915
14	Fine motor	Q4 vs Q1-Q2	186	0.13 (-0.22, 0.47)	.469	-0.04 (-0.39, 0.31)	.830	.953
14	Receptive language	Q3 vs Q1-Q2	186	-0.29 (-0.64, 0.07)	.116	-0.39 (-0.73, -0.05)	.026	.423
14	Receptive language	Q4 vs Q1-Q2	186	0.14 (-0.19, 0.48)	.399	0.15 (-0.22, 0.51)	.427	.915
14	Expressive language	Q3 vs Q1-Q2	186	0.12 (-0.24, 0.47)	.516	0.06 (-0.29, 0.40)	.752	.915
14	Expressive language	Q4 vs Q1-Q2	186	0.23 (-0.11, 0.57)	.189	0.15 (-0.22, 0.53)	.417	.915
18	Gross motor	Q3 vs Q1-Q2	190	0.05 (-0.28, 0.38)	.771	-0.02 (-0.33, 0.30)	.914	.953
18	Gross motor	Q4 vs Q1-Q2	190	-0.02 (-0.32, 0.29)	.901	-0.06 (-0.37, 0.25)	.692	.915
18	Visual reception	Q3 vs Q1-Q2	190	0.13 (-0.21, 0.48)	.447	0.10 (-0.24, 0.45)	.561	.915
18	Visual reception	Q4 vs Q1-Q2	190	0.10 (-0.20, 0.39)	.524	0.06 (-0.23, 0.35)	.692	.915
18	Fine motor	Q3 vs Q1-Q2	190	0.13 (-0.20, 0.46)	.432	0.12 (-0.19, 0.42)	.461	.915
18	Fine motor	Q4 vs Q1-Q2	190	0.06 (-0.28, 0.40)	.729	-0.01 (-0.36, 0.35)	.977	.997
18	Receptive language	Q3 vs Q1-Q2	190	0.44 (0.05, 0.83)	.029	0.35 (-0.02, 0.72)	.065	.539
18	Receptive language	Q4 vs Q1-Q2	190	0.09 (-0.24, 0.41)	.608	0.15 (-0.17, 0.47)	.366	.915
18	Expressive language	Q3 vs Q1-Q2	190	0.20 (-0.13, 0.53)	.236	0.14 (-0.19, 0.46)	.415	.915
18	Expressive language	Q4 vs Q1-Q2	190	0.04 (-0.30, 0.37)	.828	0.02 (-0.33, 0.37)	.904	.953
24	Gross motor	Q3 vs Q1-Q2	189	0.05 (-0.33, 0.43)	.796	-0.03 (-0.41, 0.34)	.864	.953
24	Gross motor	Q4 vs Q1-Q2	189	-0.05 (-0.40, 0.31)	.803	-0.12 (-0.47, 0.23)	.496	.915

© 2026 Nagata A et al. *JAMA Network Open*.

24	Visual reception	Q3 vs Q1-Q2	189	-0.16 (-0.51, 0.18)	.352	-0.28 (-0.60, 0.05)	.098	.702
24	Visual reception	Q4 vs Q1-Q2	189	-0.19 (-0.51, 0.14)	.257	-0.23 (-0.55, 0.09)	.165	.915
24	Fine motor	Q3 vs Q1-Q2	189	-0.14 (-0.52, 0.25)	.485	-0.25 (-0.61, 0.12)	.192	.915
24	Fine motor	Q4 vs Q1-Q2	189	-0.40 (-0.68, -0.11)	.007	-0.35 (-0.65, -0.05)	.022	.423
24	Receptive language	Q3 vs Q1-Q2	189	0.17 (-0.15, 0.50)	.296	0.08 (-0.24, 0.40)	.626	.915
24	Receptive language	Q4 vs Q1-Q2	189	-0.03 (-0.36, 0.29)	.849	-0.02 (-0.33, 0.29)	.915	.953
24	Expressive language	Q3 vs Q1-Q2	189	0.13 (-0.19, 0.46)	.421	0.05 (-0.27, 0.36)	.769	.915
24	Expressive language	Q4 vs Q1-Q2	189	-0.10 (-0.40, 0.21)	.538	-0.11 (-0.43, 0.21)	.494	.915
32	Gross motor	Q3 vs Q1-Q2	185	0.12 (-0.24, 0.48)	.499	0.08 (-0.28, 0.44)	.674	.915
32	Gross motor	Q4 vs Q1-Q2	185	-0.03 (-0.38, 0.32)	.854	-0.07 (-0.41, 0.28)	.711	.915
32	Visual reception	Q3 vs Q1-Q2	185	-0.08 (-0.43, 0.26)	.627	-0.10 (-0.44, 0.23)	.552	.915
32	Visual reception	Q4 vs Q1-Q2	185	-0.23 (-0.52, 0.07)	.132	-0.17 (-0.46, 0.13)	.268	.915
32	Fine motor	Q3 vs Q1-Q2	185	0.02 (-0.32, 0.36)	.909	-0.00 (-0.34, 0.34)	.997	.997
32	Fine motor	Q4 vs Q1-Q2	185	-0.39 (-0.71, -0.06)	.020	-0.33 (-0.67, 0.00)	.052	.525
32	Receptive language	Q3 vs Q1-Q2	185	-0.01 (-0.33, 0.32)	.957	0.02 (-0.30, 0.33)	.907	.953
32	Receptive language	Q4 vs Q1-Q2	185	-0.08 (-0.37, 0.22)	.606	0.04 (-0.24, 0.33)	.755	.915
32	Expressive language	Q3 vs Q1-Q2	185	0.06 (-0.30, 0.42)	.760	0.07 (-0.28, 0.42)	.686	.915
32	Expressive language	Q4 vs Q1-Q2	185	-0.12 (-0.48, 0.24)	.515	-0.08 (-0.46, 0.30)	.666	.915

Abbreviations: DIN, dinotefuran; dm-ACE, desmethyl-acetamiprid; FDR, false discovery rate; MSEL, Mullen Scales of Early Learning; Q, quartile

[a] Adjusted models mutually included DIN and dm-ACE and were adjusted for exact age at assessment, child sex, maternal education, paternal education, and preterm birth. Standard errors were clustered at the maternal level. Mean differences are in standardized MSEL score units. Negative values indicate lower scores in the higher concentration group relative to the combined Q1-Q2 reference.

[b] FDR *q* values use the Benjamini-Hochberg procedure across 50 adjusted tests within the biomarker family.

© 2026 Nagata A et al. *JAMA Network Open*.

eTable 5. Associations of Cord Serum dm-ACE Concentration Groups With MSEL Domain Scores

Age, mo	MSEL domain	Comparison	Participants, No.	Crude mean difference (95% CI)	*P* value	Adjusted mean difference (95% CI)[a]	*P* value	FDR *q* value[b]
10	Gross motor	Q3 vs Q1-Q2	195	-0.09 (-0.37, 0.19)	.532	-0.17 (-0.43, 0.10)	.214	.761
10	Gross motor	Q4 vs Q1-Q2	195	-0.27 (-0.59, 0.06)	.105	-0.20 (-0.50, 0.10)	.196	.756
10	Visual reception	Q3 vs Q1-Q2	195	-0.08 (-0.44, 0.29)	.685	-0.12 (-0.48, 0.24)	.514	.866
10	Visual reception	Q4 vs Q1-Q2	195	-0.46 (-0.81, -0.11)	.011	-0.36 (-0.69, -0.03)	.033	.722
10	Fine motor	Q3 vs Q1-Q2	195	-0.10 (-0.44, 0.24)	.573	-0.13 (-0.47, 0.20)	.440	.866
10	Fine motor	Q4 vs Q1-Q2	195	-0.52 (-0.85, -0.19)	.002	-0.46 (-0.79, -0.14)	.005	.273
10	Receptive language	Q3 vs Q1-Q2	195	-0.06 (-0.39, 0.27)	.707	-0.10 (-0.42, 0.22)	.537	.866
10	Receptive language	Q4 vs Q1-Q2	195	-0.24 (-0.59, 0.11)	.181	-0.14 (-0.48, 0.20)	.418	.866
10	Expressive language	Q3 vs Q1-Q2	195	0.01 (-0.26, 0.28)	.945	-0.04 (-0.30, 0.23)	.789	.992
10	Expressive language	Q4 vs Q1-Q2	195	-0.11 (-0.43, 0.21)	.492	-0.06 (-0.37, 0.25)	.717	.992
14	Gross motor	Q3 vs Q1-Q2	186	0.13 (-0.22, 0.48)	.470	0.05 (-0.29, 0.40)	.767	.992
14	Gross motor	Q4 vs Q1-Q2	186	-0.05 (-0.42, 0.33)	.812	-0.00 (-0.36, 0.36)	.992	.992
14	Visual reception	Q3 vs Q1-Q2	186	0.36 (0.04, 0.68)	.027	0.28 (-0.03, 0.59)	.075	.756
14	Visual reception	Q4 vs Q1-Q2	186	-0.01 (-0.38, 0.36)	.947	0.04 (-0.31, 0.38)	.835	.992
14	Fine motor	Q3 vs Q1-Q2	186	0.23 (-0.11, 0.57)	.182	0.16 (-0.18, 0.49)	.356	.866
14	Fine motor	Q4 vs Q1-Q2	186	0.30 (-0.05, 0.64)	.093	0.29 (-0.05, 0.63)	.091	.756
14	Receptive language	Q3 vs Q1-Q2	186	-0.04 (-0.41, 0.33)	.850	-0.12 (-0.48, 0.24)	.522	.866
14	Receptive language	Q4 vs Q1-Q2	186	0.09 (-0.25, 0.43)	.615	0.12 (-0.20, 0.44)	.473	.866
14	Expressive language	Q3 vs Q1-Q2	186	0.12 (-0.24, 0.49)	.514	0.03 (-0.33, 0.39)	.874	.992
14	Expressive language	Q4 vs Q1-Q2	186	-0.02 (-0.40, 0.36)	.922	-0.02 (-0.36, 0.33)	.930	.992
18	Gross motor	Q3 vs Q1-Q2	190	-0.10 (-0.40, 0.20)	.502	-0.11 (-0.38, 0.17)	.456	.866
18	Gross motor	Q4 vs Q1-Q2	190	-0.29 (-0.61, 0.04)	.084	-0.17 (-0.50, 0.15)	.289	.761
18	Visual reception	Q3 vs Q1-Q2	190	0.21 (-0.10, 0.53)	.184	0.21 (-0.11, 0.53)	.194	.756
18	Visual reception	Q4 vs Q1-Q2	190	-0.03 (-0.35, 0.30)	.871	0.04 (-0.28, 0.35)	.815	.992
18	Fine motor	Q3 vs Q1-Q2	190	0.32 (0.00, 0.63)	.049	0.32 (0.01, 0.64)	.043	.722
18	Fine motor	Q4 vs Q1-Q2	190	0.16 (-0.19, 0.52)	.370	0.25 (-0.10, 0.59)	.163	.756
18	Receptive language	Q3 vs Q1-Q2	190	0.29 (-0.08, 0.67)	.122	0.32 (-0.05, 0.69)	.090	.756
18	Receptive language	Q4 vs Q1-Q2	190	-0.28 (-0.62, 0.05)	.100	-0.18 (-0.49, 0.13)	.265	.761
18	Expressive language	Q3 vs Q1-Q2	190	0.27 (-0.09, 0.63)	.137	0.29 (-0.07, 0.65)	.120	.756
18	Expressive language	Q4 vs Q1-Q2	190	-0.06 (-0.38, 0.26)	.703	0.03 (-0.29, 0.34)	.864	.992
24	Gross motor	Q3 vs Q1-Q2	189	-0.25 (-0.59, 0.09)	.155	-0.26 (-0.60, 0.09)	.143	.756
24	Gross motor	Q4 vs Q1-Q2	189	-0.35 (-0.73, 0.04)	.076	-0.26 (-0.64, 0.12)	.173	.756

© 2026 Nagata A et al. *JAMA Network Open*.

24	Visual reception	Q3 vs Q1-Q2	189	0.20 (-0.13, 0.54)	.240	0.18 (-0.15, 0.52)	.287	.761
24	Visual reception	Q4 vs Q1-Q2	189	-0.12 (-0.44, 0.21)	.484	-0.00 (-0.29, 0.29)	.978	.992
24	Fine motor	Q3 vs Q1-Q2	189	0.16 (-0.17, 0.48)	.343	0.17 (-0.14, 0.48)	.285	.761
24	Fine motor	Q4 vs Q1-Q2	189	-0.13 (-0.47, 0.20)	.427	-0.04 (-0.34, 0.27)	.814	.992
24	Receptive language	Q3 vs Q1-Q2	189	0.09 (-0.24, 0.42)	.590	0.09 (-0.24, 0.42)	.595	.930
24	Receptive language	Q4 vs Q1-Q2	189	0.03 (-0.29, 0.36)	.836	0.12 (-0.17, 0.42)	.414	.866
24	Expressive language	Q3 vs Q1-Q2	189	0.12 (-0.19, 0.44)	.447	0.12 (-0.19, 0.44)	.447	.866
24	Expressive language	Q4 vs Q1-Q2	189	0.09 (-0.24, 0.43)	.581	0.18 (-0.14, 0.51)	.268	.761
32	Gross motor	Q3 vs Q1-Q2	185	-0.24 (-0.57, 0.09)	.161	-0.24 (-0.56, 0.08)	.147	.756
32	Gross motor	Q4 vs Q1-Q2	185	-0.09 (-0.45, 0.28)	.647	0.02 (-0.32, 0.36)	.919	.992
32	Visual reception	Q3 vs Q1-Q2	185	0.10 (-0.22, 0.42)	.533	0.13 (-0.19, 0.44)	.430	.866
32	Visual reception	Q4 vs Q1-Q2	185	0.04 (-0.29, 0.37)	.807	0.10 (-0.19, 0.39)	.495	.866
32	Fine motor	Q3 vs Q1-Q2	185	-0.08 (-0.42, 0.25)	.624	-0.05 (-0.38, 0.27)	.759	.992
32	Fine motor	Q4 vs Q1-Q2	185	-0.13 (-0.49, 0.23)	.470	-0.06 (-0.38, 0.26)	.718	.992
32	Receptive language	Q3 vs Q1-Q2	185	-0.03 (-0.34, 0.28)	.845	0.00 (-0.30, 0.30)	.989	.992
32	Receptive language	Q4 vs Q1-Q2	185	-0.11 (-0.44, 0.22)	.501	-0.07 (-0.37, 0.23)	.643	.975
32	Expressive language	Q3 vs Q1-Q2	185	0.05 (-0.34, 0.43)	.808	0.06 (-0.33, 0.44)	.765	.992
32	Expressive language	Q4 vs Q1-Q2	185	-0.04 (-0.41, 0.32)	.820	-0.00 (-0.35, 0.34)	.981	.992

Abbreviations: CI, confidence interval; DIN, dinotefuran; dm-ACE, desmethyl-acetamiprid; FDR, false discovery rate; MSEL, Mullen Scales of Early Learning; Q, quartile. Mean differences are expressed in standardized MSEL score units; negative values indicate lower scores relative to the combined Q1–Q2 reference group.

[a] Adjusted models mutually included DIN and dm-ACE and additionally included exact assessment age, child sex, maternal education, paternal education, and preterm birth. Standard errors were clustered at the maternal level.

[b] FDR *q* values were calculated for the 50 adjusted dm-ACE comparisons using the Benjamini–Hochberg procedure.

© 2026 Nagata A et al. *JAMA Network Open*.

eTable 6. Overall and Age-Specific GEE Associations of Cord Serum DIN and dm-ACE With MSEL Domain Scores

Panel A. Overall associations and omnibus concentration-by-age interaction tests

MSEL domain	Biomarker	Q3 vs Q1–Q2, mean difference (95% CI)	*P* value	Q4 vs Q1–Q2, mean difference (95% CI)	*P* value	Interaction χ^2 (df)	*P* value
Gross motor	DIN	−0.12 (−0.35, 0.10)	.28	−0.04 (−0.27, 0.18)	.71	16.14 (8)	.04
Gross motor	dm-ACE	−0.14 (−0.35, 0.07)	.19	−0.11 (−0.34, 0.12)	.34	7.03 (8)	.53
Visual reception	DIN	−0.08 (−0.29, 0.14)	.48	−0.06 (−0.25, 0.12)	.52	9.86 (8)	.28
Visual reception	dm-ACE	0.13 (−0.07, 0.34)	.21	−0.03 (−0.21, 0.15)	.76	10.06 (8)	.26
Fine motor	DIN	0.06 (−0.16, 0.28)	.59	−0.11 (−0.32, 0.09)	.27	13.45 (8)	.10
Fine motor	dm-ACE	0.10 (−0.11, 0.32)	.33	0.00 (−0.20, 0.21)	.97	18.85 (8)	.02
Receptive language	DIN	0.08 (−0.14, 0.30)	.49	0.10 (−0.10, 0.30)	.34	19.71 (8)	.01
Receptive language	dm-ACE	0.07 (−0.17, 0.32)	.56	0.01 (−0.20, 0.21)	.95	16.17 (8)	.04
Expressive language	DIN	0.06 (−0.17, 0.30)	.61	0.05 (−0.18, 0.28)	.69	8.12 (8)	.42
Expressive language	dm-ACE	0.18 (−0.12, 0.48)	.25	0.08 (−0.16, 0.31)	.51	4.92 (8)	.77

Panel B. Age-specific contrasts for biomarker-domain pairs with nominal omnibus interaction *P*<.05

MSEL domain	Biomarker	Age, mo	Q3 vs Q1–Q2, mean difference (95% CI)	*P* value	Q4 vs Q1–Q2, mean difference (95% CI)	*P* value
Gross motor	DIN	10	−0.31 (−0.61, −0.01)	.04	0.20 (−0.07, 0.48)	.15
Gross motor	DIN	14	−0.41 (−0.78, −0.04)	.03	−0.17 (−0.50, 0.16)	.30
Gross motor	DIN	18	0.01 (−0.29, 0.32)	.93	−0.05 (−0.34, 0.25)	.76
Gross motor	DIN	24	−0.02 (−0.39, 0.35)	.92	−0.08 (−0.43, 0.27)	.67
Gross motor	DIN	32	0.12 (−0.23, 0.47)	.51	−0.12 (−0.47, 0.24)	.51

© 2026 Nagata A et al. *JAMA Network Open*.

MSEL domain	Biomarker	Age, mo	Q3 vs Q1–Q2, mean difference (95% CI)	*P* value	Q4 vs Q1–Q2, mean difference (95% CI)	*P* value
Fine motor	dm-ACE	10	−0.09 (−0.43, 0.25)	.61	−0.44 (−0.77, −0.11)	.01
Fine motor	dm-ACE	14	0.27 (−0.06, 0.61)	.11	0.32 (−0.03, 0.66)	.07
Fine motor	dm-ACE	18	0.34 (0.03, 0.65)	.03	0.22 (−0.12, 0.57)	.20
Fine motor	dm-ACE	24	0.07 (−0.25, 0.40)	.67	−0.01 (−0.32, 0.29)	.93
Fine motor	dm-ACE	32	−0.07 (−0.39, 0.25)	.67	−0.07 (−0.40, 0.26)	.69
Receptive language	DIN	10	0.25 (−0.07, 0.57)	.13	0.24 (−0.09, 0.57)	.15
Receptive language	DIN	14	−0.35 (−0.69, −0.01)	.05	0.11 (−0.22, 0.45)	.51
Receptive language	DIN	18	0.42 (0.05, 0.78)	.03	0.16 (−0.15, 0.47)	.30
Receptive language	DIN	24	0.14 (−0.17, 0.44)	.39	−0.01 (−0.32, 0.29)	.93
Receptive language	DIN	32	−0.07 (−0.38, 0.24)	.66	0.00 (−0.27, 0.27)	.99
Receptive language	dm-ACE	10	−0.03 (−0.36, 0.30)	.87	−0.13 (−0.46, 0.21)	.45
Receptive language	dm-ACE	14	−0.02 (−0.40, 0.36)	.91	0.17 (−0.15, 0.48)	.29
Receptive language	dm-ACE	18	0.32 (−0.07, 0.70)	.11	−0.14 (−0.45, 0.17)	.38
Receptive language	dm-ACE	24	0.12 (−0.21, 0.45)	.47	0.15 (−0.15, 0.44)	.34
Receptive language	dm-ACE	32	−0.01 (−0.33, 0.31)	.94	0.00 (−0.31, 0.30)	.98

Abbreviations: CI, confidence interval; DIN, dinotefuran; dm-ACE, desmethyl-acetamiprid; GEE, generalized estimating equation; MSEL, Mullen Scales of Early Learning; Q, quartile.

Mean differences are expressed in standardized MSEL score units; negative values indicate lower scores relative to the combined Q1–Q2 reference. GEE models used a Gaussian distribution, identity link, exchangeable working correlation, and maternal-level cluster-robust standard errors. Models mutually included DIN and dm-ACE and were adjusted for categorical assessment age, exact age at assessment, child sex, maternal education, paternal education, and preterm birth. Overall estimates represent average associations across assessments at 10, 14, 18, 24, and 32 months. Omnibus tests jointly evaluated the 8 concentration-group-by-age interaction terms. Panel B presents age-specific contrasts only for concentration-domain pairs with nominal omnibus interaction *P*<.05. No additional multiplicity correction was applied to these supplementary analyses.

Models included 200 children from 197 maternal clusters. Repeated observations were 974 for gross motor, 988 for fine motor, 988 for visual reception, 968 for receptive language, and 980 for expressive language, out of a maximum of 1000 observations per domain

© 2026 Nagata A et al. *JAMA Network Open*.

eTable 7. Associations of Cord Serum DIN and dm-ACE With Age-Specific Standardized MSEL Domain Scores After Additional Adjustment for Maternal Smoking and Alcohol Use During Pregnancy

MSEL domain	Concentration group	DIN, mean difference (95% CI)	*P* value	dm-ACE, mean difference (95% CI)	*P* value
**MSEL at 10 months (n=195)**					
Gross motor	Q3	−0.36 (−0.66 to −0.07)	.02	−0.18 (−0.44 to 0.08)	.17
	Q4	0.07 (−0.22 to 0.36)	.65	−0.20 (−0.50 to 0.10)	.19
Visual reception	Q3	−0.06 (−0.42 to 0.30)	.73	−0.12 (−0.48 to 0.24)	.51
	Q4	−0.09 (−0.46 to 0.27)	.63	−0.36 (−0.69 to −0.02)	.04
Fine motor	Q3	0.05 (−0.27 to 0.38)	.75	−0.14 (−0.48 to 0.19)	.41
	Q4	0.06 (−0.27 to 0.39)	.72	−0.46 (−0.79 to −0.14)	.005
Receptive language	Q3	0.20 (−0.14 to 0.53)	.25	−0.10 (−0.42 to 0.22)	.54
	Q4	0.15 (−0.18 to 0.48)	.36	−0.14 (−0.48 to 0.20)	.43
Expressive language	Q3	−0.06 (−0.35 to 0.24)	.70	−0.04 (−0.30 to 0.23)	.80
	Q4	0.15 (−0.15 to 0.44)	.34	−0.06 (−0.37 to 0.25)	.71
**MSEL at 14 months (n=186)**					
Gross motor	Q3	−0.42 (−0.79 to −0.04)	.03	0.02 (−0.33 to 0.37)	.91
	Q4	−0.21 (−0.57 to 0.15)	.26	−0.01 (−0.36 to 0.34)	.97
Visual reception	Q3	−0.26 (−0.63 to 0.10)	.16	0.24 (−0.06 to 0.55)	.12
	Q4	−0.12 (−0.43 to 0.19)	.45	0.02 (−0.32 to 0.37)	.89
Fine motor	Q3	0.16 (−0.14 to 0.47)	.29	0.13 (−0.20 to 0.46)	.45
	Q4	−0.06 (−0.41 to 0.29)	.75	0.28 (−0.05 to 0.62)	.10
Receptive language	Q3	−0.39 (−0.73 to −0.05)	.03	−0.14 (−0.50 to 0.23)	.46
	Q4	0.14 (−0.22 to 0.50)	.46	0.11 (−0.20 to 0.43)	.48
Expressive language	Q3	0.07 (−0.28 to 0.41)	.71	0.04 (−0.32 to 0.40)	.83
	Q4	0.17 (−0.20 to 0.53)	.37	−0.02 (−0.37 to 0.33)	.93
**MSEL at 18 months (n=190)**					
Gross motor	Q3	−0.02 (−0.34 to 0.29)	.88	−0.12 (−0.40 to 0.15)	.38
	Q4	−0.07 (−0.38 to 0.23)	.63	−0.17 (−0.50 to 0.15)	.29
Visual reception	Q3	0.10 (−0.24 to 0.45)	.56	0.20 (−0.12 to 0.53)	.21

© 2026 Nagata A et al. *JAMA Network Open*.

MSEL domain	Concentration group	DIN, mean difference (95% CI)	*P* value	dm-ACE, mean difference (95% CI)	*P* value
	Q4	0.06 (−0.24 to 0.35)	.70	0.04 (−0.28 to 0.35)	.82
Fine motor	Q3	0.11 (−0.20 to 0.42)	.49	0.31 (0.00 to 0.63)	.05
	Q4	−0.02 (−0.37 to 0.34)	.93	0.25 (−0.10 to 0.59)	.16
Receptive language	Q3	0.34 (−0.03 to 0.70)	.07	0.31 (−0.06 to 0.68)	.10
	Q4	0.13 (−0.19 to 0.45)	.42	−0.17 (−0.48 to 0.14)	.27
Expressive language	Q3	0.13 (−0.20 to 0.45)	.44	0.29 (−0.08 to 0.65)	.12
	Q4	0.01 (−0.34 to 0.36)	.95	0.03 (−0.29 to 0.35)	.86
**MSEL at 24 months (n=189)**					
Gross motor	Q3	−0.04 (−0.41 to 0.34)	.85	−0.28 (−0.63 to 0.07)	.11
	Q4	−0.13 (−0.48 to 0.21)	.45	−0.27 (−0.65 to 0.11)	.16
Visual reception	Q3	−0.27 (−0.61 to 0.06)	.11	0.19 (−0.15 to 0.53)	.29
	Q4	−0.23 (−0.54 to 0.09)	.17	0.00 (−0.29 to 0.29)	.98
Fine motor	Q3	−0.24 (−0.61 to 0.13)	.21	0.16 (−0.15 to 0.48)	.30
	Q4	−0.34 (−0.64 to −0.03)	.03	−0.04 (−0.34 to 0.26)	.80
Receptive language	Q3	0.08 (−0.25 to 0.41)	.63	0.08 (−0.24 to 0.41)	.62
	Q4	−0.02 (−0.32 to 0.28)	.91	0.12 (−0.18 to 0.42)	.42
Expressive language	Q3	0.05 (−0.26 to 0.37)	.74	0.14 (−0.18 to 0.46)	.40
	Q4	−0.09 (−0.42 to 0.23)	.57	0.19 (−0.14 to 0.52)	.26
**MSEL at 32 months (n=185)**					
Gross motor	Q3	0.11 (−0.25 to 0.46)	.55	−0.25 (−0.57 to 0.07)	.13
	Q4	−0.04 (−0.39 to 0.31)	.82	−0.01 (−0.35 to 0.32)	.95
Visual reception	Q3	−0.13 (−0.47 to 0.21)	.46	0.13 (−0.18 to 0.44)	.40
	Q4	−0.19 (−0.47 to 0.10)	.20	0.13 (−0.16 to 0.41)	.39
Fine motor	Q3	0.02 (−0.32 to 0.35)	.93	−0.06 (−0.38 to 0.26)	.71
	Q4	−0.32 (−0.65 to 0.01)	.06	−0.08 (−0.40 to 0.24)	.64
Receptive language	Q3	0.01 (−0.31 to 0.33)	.97	0.00 (−0.30 to 0.30)	>.99
	Q4	0.03 (−0.26 to 0.32)	.84	−0.06 (−0.36 to 0.23)	.68
Expressive language	Q3	0.06 (−0.28 to 0.41)	.72	0.04 (−0.35 to 0.43)	.84

© 2026 Nagata A et al. *JAMA Network Open*.

MSEL domain	Concentration group	DIN, mean difference (95% CI)	*P* value	dm-ACE, mean difference (95% CI)	*P* value
	Q4	−0.11 (−0.48 to 0.27)	.59	−0.01 (−0.34 to 0.33)	.97

Abbreviations: CI, confidence interval; DIN, dinotefuran; dm-ACE, desmethyl-acetamiprid; GSEM, generalized structural equation model; MSEL, Mullen Scales of Early Learning; Q, quartile.
Q1–Q2 was the combined reference group. Mean differences are expressed in standardized MSEL score units; negative values indicate lower scores relative to the reference group.
At each assessment age, the 5 MSEL domains were jointly modeled using GSEM. Models included DIN and dm-ACE simultaneously and were adjusted for exact age at assessment, child sex, maternal and paternal education, preterm birth, maternal smoking during pregnancy, and maternal alcohol use after 12 weeks of pregnancy. Maternal-level cluster-robust standard errors were used.
P values are 2-sided and nominal. No additional multiplicity correction was applied because this was a sensitivity analysis; false discovery rate–adjusted results for the primary models are reported in eTables 4 and 5.

© 2026 Nagata A et al. *JAMA Network Open*.

eTable 8. Associations of Cord Serum DIN and dm-ACE With WISC-IV Indices and Full-Scale IQ at 9 Years of Age

DIN (WISC-IV outcome)	Comparison	Crude mean difference (95% CI)	*P* value	Adjusted mean difference (95% CI)	*P* value	FDR *q* value
Verbal Comprehension Index	Q3 vs Q1–Q2	−0.34 (−0.67 to −0.02)	.03	−0.29 (−0.60 to 0.02)	.07	.11
	Q4 vs Q1–Q2	−0.31 (−0.62 to 0.00)	.05	−0.24 (−0.57 to 0.09)	.15	.17
Perceptual Reasoning Index	Q3 vs Q1–Q2	−0.27 (−0.63 to 0.09)	.14	−0.26 (−0.62 to 0.10)	.15	.17
	Q4 vs Q1–Q2	−0.39 (−0.74 to −0.04)	.02	−0.39 (−0.72 to −0.05)	.02	.05
Working Memory Index	Q3 vs Q1–Q2	−0.45 (−0.80 to −0.11)	.01	−0.42 (−0.78 to −0.05)	.02	.05
	Q4 vs Q1–Q2	−0.31 (−0.64 to 0.03)	.07	−0.23 (−0.59 to 0.13)	.20	.20
Processing Speed Index	Q3 vs Q1–Q2	−0.47 (−0.77 to −0.16)	.001	−0.50 (−0.81 to −0.20)	.01	.013
	Q4 vs Q1–Q2	−0.38 (−0.69 to −0.07)	.01	−0.26 (−0.57 to 0.05)	.10	.15
Full-Scale IQ	Q3 vs Q1–Q2	−0.49 (−0.82 to −0.16)	.005	−0.46 (−0.78 to −0.15)	.01	.023
	Q4 vs Q1–Q2	−0.46 (−0.77 to −0.15)	.01	−0.37 (−0.69 to −0.06)	.02	.05
**dm-ACE (WISC-IV outcome)**						
Verbal Comprehension Index	Q3 vs Q1–Q2	−0.06 (−0.39 to 0.26)	.70	−0.05 (−0.36 to 0.26)	.73	.73
	Q4 vs Q1–Q2	−0.24 (−0.55 to 0.07)	.13	−0.30 (−0.59 to −0.01)	.04	.11
Perceptual Reasoning Index	Q3 vs Q1–Q2	−0.28 (−0.63 to 0.08)	.12	−0.23 (−0.58 to 0.12)	.19	.31
	Q4 vs Q1–Q2	−0.42 (−0.77 to −0.07)	.006	−0.47 (−0.81 to −0.14)	.01	.031
Working Memory Index	Q3 vs Q1–Q2	−0.12 (−0.45 to 0.22)	.49	−0.12 (−0.46 to 0.22)	.48	.53
	Q4 vs Q1–Q2	−0.22 (−0.58 to 0.14)	.23	−0.27 (−0.63 to 0.10)	.15	.30
Processing Speed Index	Q3 vs Q1–Q2	−0.18 (−0.48 to 0.12)	.24	−0.16 (−0.45 to 0.14)	.29	.36
	Q4 vs Q1–Q2	−0.31 (−0.65 to 0.03)	.07	−0.37 (−0.68 to −0.06)	.02	.07
Full-Scale IQ	Q3 vs Q1–Q2	−0.21 (−0.53 to 0.11)	.20	−0.18 (−0.49 to 0.13)	.24	.34
	Q4 vs Q1–Q2	−0.39 (−0.72 to −0.06)	.002	−0.46 (−0.74 to −0.17)	.01	.020

Abbreviations: CI, confidence interval; DIN, dinotefuran; dm-ACE, desmethyl-acetamiprid; FDR, false discovery rate; FSIQ, Full-Scale IQ; Q, quartile; WISC-IV, Wechsler Intelligence Scale for Children–Fourth Edition.

Q1–Q2 was the combined reference group. Mean differences are expressed in standardized WISC-IV score units; negative values indicate lower scores relative to the reference group. P values are 2-sided.

Crude models included 1 exposure at a time. Adjusted models included DIN and dm-ACE simultaneously and were adjusted for exact age at assessment, child sex, maternal and paternal education, and preterm birth. Maternal-level cluster-robust standard errors were used.

FDR *q* values were calculated using the Benjamini–Hochberg procedure separately for the 10 adjusted comparisons within each exposure family (5 outcomes × 2 concentration-group comparisons). The 4 WISC-IV indices were the principal middle-childhood outcomes; FSIQ was a complementary outcome.

eTable 9. Associations of Cord Serum DIN and dm-ACE With WISC-IV Indices and Full-Scale IQ After Additional Adjustment for Maternal Smoking and Alcohol Use During Pregnancy

© 2026 Nagata A et al. *JAMA Network Open*.

WISC-IV outcome	Concentration group	DIN, mean difference (95% CI)	*P* value	dm-ACE, mean difference (95% CI)	*P* value
Verbal Comprehension Index	Q3	−0.31 (−0.63 to 0.01)	.05	−0.06 (−0.37 to 0.25)	.69
	Q4	−0.26 (−0.61 to 0.08)	.13	−0.29 (−0.58 to 0.00)	.04
Perceptual Reasoning Index	Q3	−0.29 (−0.65 to 0.07)	.11	−0.26 (−0.60 to 0.09)	.14
	Q4	−0.41 (−0.74 to −0.08)	.01	−0.47 (−0.80 to −0.14)	.01
Working Memory Index	Q3	−0.43 (−0.79 to −0.06)	.02	−0.13 (−0.47 to 0.21)	.44
	Q4	−0.24 (−0.60 to 0.12)	.18	−0.27 (−0.63 to 0.10)	.15
Processing Speed Index	Q3	−0.52 (−0.83 to −0.22)	<.001	−0.17 (−0.47 to 0.13)	.27
	Q4	−0.28 (−0.58 to 0.03)	.07	−0.37 (−0.68 to −0.05)	.02
Full-Scale IQ	Q3	−0.49 (−0.81 to −0.17)	.01	−0.20 (−0.51 to 0.11)	.19
	Q4	−0.40 (−0.71 to −0.09)	.01	−0.45 (−0.74 to −0.17)	.01

Abbreviations: CI, confidence interval; DIN, dinotefuran; dm-ACE, desmethyl-acetamiprid; FSIQ, Full-Scale IQ; Q, quartile; WISC-IV, Wechsler Intelligence Scale for Children–Fourth Edition.

Q1–Q2 was the combined reference group. Mean differences are expressed in standardized WISC-IV score units; negative values indicate lower scores relative to the reference group. P values are 2-sided and nominal.

Models included DIN and dm-ACE simultaneously and were adjusted for exact age at assessment, child sex, maternal and paternal education, preterm birth, maternal smoking during pregnancy, and maternal alcohol use after 12 weeks of pregnancy. Maternal-level cluster-robust standard errors were used.

No additional multiplicity correction was applied because this was a sensitivity analysis; FDR-adjusted results for the primary models are reported in eTable 8. The 4 WISC-IV indices were the principal middle-childhood outcomes; FSIQ was a complementary outcome.

© 2026 Nagata A et al. *JAMA Network Open*.

eTable 10. Sensitivity Analyses of Associations of Cord Serum DIN and dm-ACE With MSEL and WISC-IV Outcomes Without Adjustment for Preterm Birth

Panel A. Age-Specific Standardized MSEL Domain Scores

MSEL domain	Concentration group	DIN, mean difference (95% CI)	*P* value	dm-ACE, mean difference (95% CI)	*P* value
**MSEL at 10 months (n=195)**					
Gross motor	Q3	−0.36 (−0.66 to −0.06)	.01	−0.17 (−0.43 to 0.10)	.22
	Q4	0.09 (−0.19 to 0.37)	.53	−0.23 (−0.53 to 0.07)	.13
Visual reception	Q3	−0.06 (−0.42 to 0.30)	.74	−0.12 (−0.48 to 0.24)	.51
	Q4	−0.07 (−0.44 to 0.29)	.69	−0.38 (−0.71 to −0.05)	.02
Fine motor	Q3	0.05 (−0.28 to 0.38)	.77	−0.13 (−0.47 to 0.20)	.43
	Q4	0.06 (−0.26 to 0.39)	.70	−0.47 (−0.79 to −0.15)	.01
Receptive language	Q3	0.20 (−0.13 to 0.54)	.24	−0.10 (−0.42 to 0.22)	.53
	Q4	0.18 (−0.15 to 0.50)	.29	−0.17 (−0.51 to 0.16)	.30
Expressive language	Q3	−0.06 (−0.36 to 0.24)	.68	−0.04 (−0.31 to 0.23)	.78
	Q4	0.15 (−0.15 to 0.45)	.31	−0.08 (−0.38 to 0.23)	.62
**MSEL at 14 months (n=186)**					
Gross motor	Q3	−0.40 (−0.78 to −0.01)	.04	0.06 (−0.29 to 0.41)	.74
	Q4	−0.16 (−0.53 to 0.21)	.39	−0.06 (−0.42 to 0.30)	.74
Visual reception	Q3	−0.27 (−0.64 to 0.10)	.15	0.29 (−0.03 to 0.60)	.07
	Q4	−0.08 (−0.41 to 0.24)	.62	−0.03 (−0.38 to 0.32)	.88
Fine motor	Q3	0.17 (−0.15 to 0.48)	.30	0.16 (−0.18 to 0.49)	.35
	Q4	−0.04 (−0.39 to 0.32)	.83	0.29 (−0.05 to 0.62)	.09
Receptive language	Q3	−0.38 (−0.72 to −0.04)	.02	−0.11 (−0.47 to 0.25)	.53
	Q4	0.16 (−0.21 to 0.54)	.39	0.07 (−0.24 to 0.39)	.64
Expressive language	Q3	0.06 (−0.29 to 0.40)	.74	0.03 (−0.33 to 0.39)	.87
	Q4	0.16 (−0.22 to 0.53)	.41	−0.02 (−0.37 to 0.33)	.90
**MSEL at 18 months (n=190)**					
Gross motor	Q3	0.01 (−0.33 to 0.35)	.96	−0.08 (−0.38 to 0.22)	.59
	Q4	0.00 (−0.31 to 0.31)	>.99	−0.24 (−0.57 to 0.10)	.16

© 2026 Nagata A et al. *JAMA Network Open*.

MSEL domain	Concentration group	DIN, mean difference (95% CI)	*P* value	dm-ACE, mean difference (95% CI)	*P* value
Visual reception	Q3	0.12 (−0.23 to 0.47)	.51	0.22 (−0.10 to 0.54)	.16
	Q4	0.09 (−0.20 to 0.39)	.52	0.00 (−0.31 to 0.32)	.98
Fine motor	Q3	0.13 (−0.19 to 0.45)	.41	0.34 (0.02 to 0.66)	.03
	Q4	0.03 (−0.31 to 0.38)	.84	0.21 (−0.15 to 0.56)	.25
Receptive language	Q3	0.36 (−0.01 to 0.73)	.05	0.33 (−0.04 to 0.71)	.08
	Q4	0.19 (−0.14 to 0.51)	.25	−0.22 (−0.53 to 0.10)	.18
Expressive language	Q3	0.14 (−0.19 to 0.47)	.39	0.29 (−0.07 to 0.65)	.11
	Q4	0.03 (−0.31 to 0.38)	.85	0.02 (−0.30 to 0.33)	.92
**MSEL at 24 months (n=189)**					
Gross motor	Q3	−0.03 (−0.41 to 0.34)	.86	−0.26 (−0.60 to 0.09)	.14
	Q4	−0.11 (−0.46 to 0.24)	.53	−0.28 (−0.65 to 0.09)	.13
Visual reception	Q3	−0.28 (−0.61 to 0.06)	.10	0.19 (−0.15 to 0.52)	.28
	Q4	−0.20 (−0.53 to 0.12)	.22	−0.04 (−0.33 to 0.25)	.78
Fine motor	Q3	−0.25 (−0.61 to 0.12)	.19	0.17 (−0.14 to 0.48)	.28
	Q4	−0.34 (−0.64 to −0.04)	.02	−0.05 (−0.35 to 0.26)	.76
Receptive language	Q3	0.08 (−0.24 to 0.40)	.62	0.09 (−0.24 to 0.42)	.58
	Q4	0.01 (−0.30 to 0.32)	.96	0.09 (−0.22 to 0.39)	.57
Expressive language	Q3	0.05 (−0.27 to 0.37)	.76	0.12 (−0.19 to 0.44)	.44
	Q4	−0.09 (−0.41 to 0.23)	.56	0.16 (−0.16 to 0.48)	.33
**MSEL at 32 months (n=185)**					
Gross motor	Q3	0.10 (−0.27 to 0.47)	.60	−0.23 (−0.56 to 0.10)	.16
	Q4	0.01 (−0.34 to 0.37)	.93	−0.06 (−0.43 to 0.30)	.73
Visual reception	Q3	−0.09 (−0.43 to 0.25)	.59	0.13 (−0.18 to 0.44)	.41
	Q4	−0.13 (−0.43 to 0.18)	.41	0.06 (−0.24 to 0.37)	.69
Fine motor	Q3	0.01 (−0.33 to 0.36)	.94	−0.05 (−0.37 to 0.28)	.78
	Q4	−0.28 (−0.63 to 0.06)	.10	−0.11 (−0.44 to 0.23)	.53
Receptive language	Q3	0.03 (−0.29 to 0.34)	.87	0.00 (−0.29 to 0.30)	.97
	Q4	0.07 (−0.22 to 0.35)	.63	−0.10 (−0.40 to 0.21)	.54

© 2026 Nagata A et al. *JAMA Network Open*.

MSEL domain	Concentration group	DIN, mean difference (95% CI)	*P* value	dm-ACE, mean difference (95% CI)	*P* value
Expressive language	Q3	0.08 (−0.27 to 0.43)	.65	0.06 (−0.32 to 0.45)	.75
	Q4	−0.06 (−0.44 to 0.33)	.77	−0.03 (−0.38 to 0.32)	.86

Panel B. Standardized WISC-IV Indices and Full-Scale IQ at age 9 Years

WISC-IV outcome	Concentration group	DIN, mean difference (95% CI)	*P* value	dm-ACE, mean difference (95% CI)	*P* value
**WISC-IV at age 9 years (n=178)**					
Verbal Comprehension Index	Q3	−0.29 (−0.61 to 0.02)	.06	−0.06 (−0.37 to 0.26)	.72
	Q4	−0.25 (−0.58 to 0.08)	.14	−0.29 (−0.57 to 0.00)	.05
Perceptual Reasoning Index	Q3	−0.26 (−0.62 to 0.10)	.16	−0.23 (−0.58 to 0.12)	.19
	Q4	−0.38 (−0.71 to −0.04)	.02	−0.48 (−0.82 to −0.15)	.01
Working Memory Index	Q3	−0.41 (−0.78 to −0.05)	.02	−0.12 (−0.45 to 0.22)	.48
	Q4	−0.23 (−0.58 to 0.13)	.20	−0.27 (−0.63 to 0.08)	.13
Processing Speed Index	Q3	−0.50 (−0.80 to −0.20)	.01	−0.15 (−0.45 to 0.14)	.30
	Q4	−0.24 (−0.55 to 0.07)	.12	−0.39 (−0.70 to −0.08)	.01
Full-Scale IQ	Q3	−0.46 (−0.78 to −0.14)	.01	−0.18 (−0.49 to 0.13)	.24
	Q4	−0.37 (−0.68 to −0.06)	.02	−0.46 (−0.75 to −0.18)	.01

Abbreviations: CI, confidence interval; DIN, dinotefuran; dm-ACE, desmethyl-acetamiprid; FSIQ, Full-Scale IQ; GSEM, generalized structural equation model; MSEL, Mullen Scales of Early Learning; Q, quartile; WISC-IV, Wechsler Intelligence Scale for Children–Fourth Edition.

Q1–Q2 was the combined reference group. Mean differences are expressed in standardized outcome-score units; negative values indicate lower scores relative to the reference group. P values are 2-sided and nominal.

At each assessment age in Panel A, the 5 MSEL domains were jointly modeled using GSEM. Outcomes in Panel B were analyzed using separate linear regression models. All models included DIN and dm-ACE simultaneously and were adjusted for exact age at assessment, child sex, and maternal and paternal education. Maternal-level cluster-robust standard errors were used.

These sensitivity models intentionally omitted preterm birth from the covariate set but retained preterm children and the same age-specific complete-case samples used in the corresponding primary models. No additional multiplicity correction was applied; primary FDR-adjusted results are reported in eTables 4, 5, and 8. FSIQ was a complementary outcome.

© 2026 Nagata A et al. *JAMA Network Open*.

eTable 11. Associations of Cord Serum DIN and dm-ACE With MSEL Early Learning Composite Scores at 10 to 32 Months

Comparison	DIN, adjusted mean difference (95% CI)	*P* value	FDR *q* value	dm-ACE, adjusted mean difference (95% CI)	*P* value	FDR q value
**MSEL Early Learning Composite at 10 months (n=195)**						
Q3 vs Q1–Q2	0.69 (−4.51 to 5.89)	.79	.96	−2.05 (−7.02 to 2.91)	.41	.69
Q4 vs Q1–Q2	1.47 (−3.42 to 6.36)	.55	.79	−5.33 (−10.15 to −0.51)	.03	.15
**MSEL Early Learning Composite at 14 months (n=188)**						
Q3 vs Q1–Q2	−2.69 (−7.31 to 1.92)	.25	.62	2.64 (−2.43 to 7.71)	.30	.69
Q4 vs Q1–Q2	−0.17 (−5.34 to 5.01)	.95	.96	2.00 (−2.24 to 6.23)	.35	.69
**MSEL Early Learning Composite at 18 months (n=191)**						
Q3 vs Q1–Q2	4.15 (−1.53 to 9.84)	.15	.62	6.52 (1.04 to 12.00)	.02	.15
Q4 vs Q1–Q2	1.51 (−3.31 to 6.33)	.53	.79	0.98 (−3.90 to 5.86)	.69	.86
**MSEL Early Learning Composite at 24 months (n=189)**						
Q3 vs Q1–Q2	−1.57 (−5.94 to 2.81)	.48	.79	3.22 (−1.24 to 7.67)	.15	.51
Q4 vs Q1–Q2	−3.75 (−7.94 to 0.43)	.07	.62	1.24 (−2.68 to 5.16)	.53	.76
**MSEL Early Learning Composite at 32 months (n=192)**						
Q3 vs Q1–Q2	−0.12 (−5.24 to 4.99)	.96	.96	0.19 (−4.96 to 5.34)	.94	.94
Q4 vs Q1–Q2	−2.81 (−7.54 to 1.92)	.24	.62	−0.33 (−4.76 to 4.10)	.88	.94

Abbreviations: CI, confidence interval; DIN, dinotefuran; dm-ACE, desmethyl-acetamiprid; ELC, Early Learning Composite; FDR, false discovery rate; MSEL, Mullen Scales of Early Learning; Q, quartile.

Q1–Q2 was the combined reference group. Mean differences are expressed in original ELC standard-score points; negative values indicate lower scores relative to the reference group. The ELC scores were calculated using the official MSEL manual procedure. P values are 2-sided.

Models included DIN and dm-ACE simultaneously and were adjusted for exact age at assessment, child sex, maternal and paternal education, and preterm birth. Maternal-level cluster-robust standard errors were used.

FDR *q* values were calculated using the Benjamini–Hochberg procedure separately for the 10 adjusted comparisons within each biomarker family (5 ages × 2 concentration-group comparisons). The ELC analysis was complementary to the primary domain-specific MSEL analyses.

© 2026 Nagata A et al. *JAMA Network Open*.

eTable 12. Trend and Continuous-Concentration Associations of Cord Serum DIN and dm-ACE With Age-Specific Standardized MSEL Domain Scores

Panel A. Trend Analysis Using Category-Specific Median $\log_2$ Concentrations

MSEL domain	DIN, adjusted mean difference (95% CI)	*P* value	dm-ACE, adjusted mean difference (95% CI)	*P* value
**MSEL at 10 months (n=195)**				
Gross motor	0.00 (−0.16 to 0.16)	.98	−0.06 (−0.16 to 0.04)	.26
Visual reception	−0.05 (−0.26 to 0.15)	.62	−0.12 (−0.23 to 0.00)	.04
Fine motor	0.03 (−0.16 to 0.23)	.74	−0.15 (−0.26 to −0.04)	.01
Receptive language	0.11 (−0.08 to 0.30)	.26	−0.05 (−0.17 to 0.06)	.36
Expressive language	0.07 (−0.10 to 0.24)	.42	−0.02 (−0.12 to 0.09)	.77
**MSEL at 14 months (n=186)**				
Gross motor	−0.15 (−0.35 to 0.05)	.13	0.01 (−0.11 to 0.13)	.88
Visual reception	−0.10 (−0.29 to 0.08)	.28	0.03 (−0.09 to 0.14)	.61
Fine motor	0.00 (−0.20 to 0.20)	.97	0.09 (−0.02 to 0.21)	.10
Receptive language	0.03 (−0.19 to 0.24)	.80	0.04 (−0.07 to 0.15)	.45
Expressive language	0.08 (−0.13 to 0.30)	.43	0.00 (−0.12 to 0.11)	.96
**MSEL at 18 months (n=190)**				
Gross motor	−0.03 (−0.21 to 0.14)	.70	−0.06 (−0.17 to 0.05)	.26
Visual reception	0.04 (−0.14 to 0.21)	.67	0.02 (−0.09 to 0.13)	.71
Fine motor	0.01 (−0.19 to 0.20)	.95	0.09 (−0.03 to 0.21)	.13
Receptive language	0.11 (−0.09 to 0.30)	.28	−0.05 (−0.16 to 0.06)	.37
Expressive language	0.02 (−0.18 to 0.22)	.84	0.02 (−0.09 to 0.12)	.73
**MSEL at 24 months (n=189)**				
Gross motor	−0.06 (−0.26 to 0.13)	.52	−0.10 (−0.22 to 0.03)	.13
Visual reception	−0.15 (−0.34 to 0.03)	.10	0.01 (−0.09 to 0.11)	.83
Fine motor	−0.21 (−0.38 to −0.04)	.01	0.00 (−0.10 to 0.10)	.97
Receptive language	0.00 (−0.18 to 0.18)	>.99	0.04 (−0.06 to 0.14)	.41
Expressive language	−0.05 (−0.24 to 0.13)	.56	0.06 (−0.05 to 0.17)	.26
**MSEL at 32 months (n=185)**				
Gross motor	−0.02 (−0.22 to 0.19)	.87	−0.01 (−0.12 to 0.10)	.88
Visual reception	−0.10 (−0.27 to 0.07)	.25	0.04 (−0.06 to 0.14)	.44
Fine motor	−0.17 (−0.37 to 0.02)	.08	−0.02 (−0.13 to 0.08)	.65
Receptive language	0.02 (−0.14 to 0.19)	.77	−0.02 (−0.12 to 0.08)	.66
Expressive language	−0.04 (−0.25 to 0.18)	.72	0.00 (−0.12 to 0.12)	.98

© 2026 Nagata A et al. *JAMA Network Open*.

Panel B. Continuous log_2 Concentration Analysis (Per Doubling)

MSEL domain	DIN, adjusted mean difference (95% CI)	*P* value	dm-ACE, adjusted mean difference (95% CI)	*P* value
**MSEL at 10 months (n=195)**				
Gross motor	0.00 (−0.15 to 0.15)	>.99	−0.07 (−0.16 to 0.02)	.13
Visual reception	−0.05 (−0.23 to 0.14)	.62	−0.10 (−0.20 to 0.00)	.04
Fine motor	0.03 (−0.14 to 0.21)	.70	−0.12 (−0.22 to −0.02)	.01
Receptive language	0.07 (−0.11 to 0.25)	.45	−0.05 (−0.16 to 0.05)	.31
Expressive language	0.05 (−0.11 to 0.21)	.55	−0.01 (−0.10 to 0.09)	.91
**MSEL at 14 months (n=186)**				
Gross motor	−0.13 (−0.32 to 0.06)	.19	0.01 (−0.11 to 0.13)	.83
Visual reception	−0.08 (−0.25 to 0.09)	.37	0.05 (−0.04 to 0.14)	.25
Fine motor	0.01 (−0.17 to 0.18)	.92	0.11 (0.03 to 0.20)	.01
Receptive language	0.00 (−0.19 to 0.20)	.96	0.05 (−0.05 to 0.15)	.36
Expressive language	0.05 (−0.14 to 0.25)	.59	0.00 (−0.10 to 0.10)	.98
**MSEL at 18 months (n=190)**				
Gross motor	−0.06 (−0.23 to 0.12)	.51	−0.05 (−0.14 to 0.05)	.36
Visual reception	0.03 (−0.13 to 0.19)	.73	0.04 (−0.05 to 0.13)	.38
Fine motor	0.01 (−0.16 to 0.18)	.87	0.10 (0.01 to 0.20)	.03
Receptive language	0.08 (−0.09 to 0.25)	.36	−0.03 (−0.12 to 0.07)	.57
Expressive language	0.00 (−0.17 to 0.18)	.95	0.04 (−0.06 to 0.13)	.45
**MSEL at 24 months (n=189)**				
Gross motor	−0.04 (−0.22 to 0.14)	.67	−0.09 (−0.20 to 0.02)	.10
Visual reception	−0.12 (−0.29 to 0.05)	.15	−0.02 (−0.11 to 0.07)	.71
Fine motor	−0.19 (−0.34 to −0.03)	.01	0.01 (−0.08 to 0.09)	.88
Receptive language	0.01 (−0.15 to 0.17)	.93	0.05 (−0.04 to 0.13)	.32
Expressive language	−0.03 (−0.20 to 0.13)	.69	0.07 (−0.02 to 0.16)	.12
**MSEL at 32 months (n=185)**				
Gross motor	−0.05 (−0.25 to 0.14)	.58	−0.01 (−0.11 to 0.10)	.90
Visual reception	−0.13 (−0.28 to 0.03)	.11	0.06 (−0.03 to 0.14)	.19
Fine motor	−0.15 (−0.33 to 0.03)	.09	0.01 (−0.08 to 0.10)	.82
Receptive language	0.01 (−0.14 to 0.15)	.93	−0.02 (−0.10 to 0.07)	.69
Expressive language	−0.09 (−0.27 to 0.10)	.35	0.03 (−0.07 to 0.13)	.54

© 2026 Nagata A et al. *JAMA Network Open*.

Abbreviations: CI, confidence interval; DIN, dinotefuran; dm-ACE, desmethyl-acetamiprid; GSEM, generalized structural equation model; MSEL, Mullen Scales of Early Learning.
Mean differences are expressed in standardized MSEL score units; negative values indicate lower scores with increasing exposure. P values are 2-sided and nominal.
At each assessment age, the 5 MSEL domains were jointly modeled using GSEM. Models included DIN and dm-ACE simultaneously and were adjusted for exact age at assessment, child sex, maternal and paternal education, and preterm birth. Maternal-level cluster-robust standard errors were used.

© 2026 Nagata A et al. *JAMA Network Open*.

eTable 13. Trend and Continuous-Concentration Associations of Cord Serum DIN and dm-ACE With WISC-IV Indices and Full-Scale IQ at Age 9 Years

Panel A. Trend Using Category-Specific Median Log_2 Concentrations

**WISC-IV outcome**	**Biomarker**	**Participants, No.**	**Adjusted mean difference (95% CI)**	***P* value**
Verbal comprehension	DIN	178	-0.16 (-0.35 to 0.03)	.10
Verbal comprehension	dm-ACE	178	-0.09 (-0.19 to 0.01)	.07
Perceptual reasoning	DIN	178	-0.23 (-0.42 to -0.04)	.02
Perceptual reasoning	dm-ACE	178	-0.16 (-0.27 to -0.05)	.006
Working memory	DIN	178	-0.17 (-0.37 to 0.03)	.10
Working memory	dm-ACE	178	-0.08 (-0.20 to 0.04)	.20
Processing speed	DIN	178	-0.19 (-0.37 to -0.01)	.04
Processing speed	dm-ACE	178	-0.11 (-0.21 to -0.01)	.04
Full-Scale IQ	DIN	178	-0.25 (-0.43 to -0.07)	.008
Full-Scale IQ	dm-ACE	178	-0.14 (-0.24 to -0.05)	.004

Panel B. Continuous Log_2 Concentrations (per doubling)

**WISC-IV outcome**	**Exposure**	**Participants, No.**	**Adjusted mean difference (95% CI)**	***P* value**
Verbal comprehension	DIN	178	-0.16 (-0.37 to 0.04)	.12
Verbal comprehension	dm-ACE	178	-0.06 (-0.15 to 0.03)	.16
Perceptual reasoning	DIN	178	-0.25 (-0.42 to -0.07)	.006
Perceptual reasoning	dm-ACE	178	-0.11 (-0.21 to -0.02)	.02
Working memory	DIN	178	-0.16 (-0.35 to 0.03)	.11
Working memory	dm-ACE	178	-0.07 (-0.18 to 0.03)	.15
Processing speed	DIN	178	-0.13 (-0.32 to 0.06)	.17
Processing speed	dm-ACE	178	-0.09 (-0.18 to -0.00)	.04
Full-Scale IQ	DIN	178	-0.23 (-0.43 to -0.04)	.02
Full-Scale IQ	dm-ACE	178	-0.11 (-0.20 to -0.03)	.01

Abbreviations: CI, confidence interval; DIN, dinotefuran; dm-ACE, desmethyl-acetamiprid; WISC-IV, Wechsler Intelligence Scale for Children–Fourth Edition.
Models mutually included DIN and dm-ACE and were adjusted for exact age at assessment, child sex, maternal education, paternal education, and preterm birth, with maternal-level cluster-robust standard errors. WISC-IV outcomes were standardized using the normative mean of 100 and SD of 15; estimates are expressed in SD units. In Panel A, each participant was assigned the median log2 concentration of their group, and the coefficient represents a 1-unit increase in the assigned log2 concentration. In Panel B, the coefficient represents a doubling of the individual measured concentration. Full-Scale IQ was a complementary outcome. No additional multiplicity correction was applied to these supporting analyses.

© 2026 Nagata A et al. *JAMA Network Open*.

eTable 14. Characteristics of Participants With and Without Complete WISC-IV Data at Age 9 Years

Characteristic	WISC-IV data available (n=178)	WISC-IV data unavailable (n=22)	Absolute standardized difference
DIN concentration, median (IQR), ng/mL	0.040 (0.021-0.060)	0.021 (0.021-0.060)	0.33
dm-ACE concentration, median (IQR), ng/mL	0.030 (0.014-0.080)	0.035 (0.014-0.080)	0.11
Maternal education, mean (SD), y	13.8 (1.8)	13.7 (1.8)	0.08
Paternal education, mean (SD), y	14.6 (2.6)	14.6 (2.5)	0.03
Male sex, No. (%)	90 (50.6)	12 (54.5)	0.08
Preterm birth (<37 wk), No. (%)	12 (6.7)	1 (4.5)	0.10
Maternal smoking during pregnancy, No. (%)	10 (5.6)	2 (9.1)	0.13
Maternal alcohol use after 12 wk, No. (%)	24 (13.5)	4 (18.2)	0.13

Abbreviations: DIN, dinotefuran; dm-ACE, desmethyl-acetamiprid; WISC-IV, Wechsler Intelligence Scale for Children-Fourth Edition.
Values are median (IQR), mean (SD), or No. (%). Absolute standardized differences for DIN and dm-ACE were calculated using log2-transformed concentrations; all other standardized differences were calculated using pooled variances. No hypothesis tests were performed because this comparison was descriptive. WISC-IV data availability was defined as complete data for the Verbal Comprehension, Perceptual Reasoning, Working Memory, and Processing Speed indices.

© 2026 Nagata A et al. *JAMA Network Open*.

eReference.

1. Nishimura T, Takei N, Tsuchiya KJ, Asano R, Mori N. Identification of neurodevelopmental trajectories in infancy and of risk factors affecting deviant development: a longitudinal birth cohort study. *Int J Epidemiol*. Apr 2016;45(2):543–53. doi:10.1093/ije/dyv363

© 2026 Nagata A et al. *JAMA Network Open*.
