# Supplementary material for: Cord Serum Neonicotinoid-Related Biomarkers and Neurodevelopment From Early to Middle Childhood
Source: JAMA Netw Open. 2026 Nov 11;9(11):e2643481. doi: 10.1001/jamanetworkopen.2026.43481 (PMC13756127; doi:10.1001/jamanetworkopen.2026.43481)
Supplement: Supplementary file 2 [file jamanetwopen-e2643481-s002.pdf]

# Data Sharing Statement

Nagata. Cord Serum Neonicotinoid-Related Biomarkers and Neurodevelopment From Early to Middle Childhood. *JAMA Netw Open*. Published November 11, 2026.
doi:10.1001/jamanetworkopen.2026.43481

**Data**
**Data available:** No

**Additional Information**
**Explanation for why data not available:** The data used in this study cannot be publicly deposited due to ethical restrictions and the legal framework governing personal information in Japan. Public sharing of datasets containing personal information is restricted under the Act on the Protection of Personal Information (Act No. 57 of May 30, 2003; amended September 9, 2015). In addition, the Ethical Guidelines for Medical and Health Research Involving Human Subjects issued by the Ministry of Education, Culture, Sports, Science and Technology and the Ministry of Health, Labour and Welfare limit open access to epidemiological data.
